# Pneumonia incidence, hospitalizations and mortality before and during the COVID-19 pandemic in Peru: An observational study

**DOI:** 10.1371/journal.pone.0351171

**Published:** 2026-10-06

**Authors:** Max Carlos Ramírez-Soto, Hugo Arroyo-Hernandez, Juan Vicente Bogado Machuca, Diego H. Stalder, Christian E. Schaerer

**Affiliations:** 1 Facultad de Ciencias de la Salud, Universidad Tecnológica del Peru, Lima, Peru; 2 Universidad de Huánuco, Huánuco, Peru; 3 Polytechnic School, National University of Asuncion, San Lorenzo, Paraguay; 4 Faculty of Engineering, National University of Asuncion, San Lorenzo, Paraguay; Monash University, AUSTRALIA

## Abstract

**Background:**

Pneumonia is a leading cause of morbidity and mortality in children and older adults. COVID-19 related disruptions in healthcare and surveillance may have altered incidence, hospitalizations, and deaths. We assessed pneumonia incidence, hospitalizations, and mortality rates in children under five and adults older than 60 years in Peru during the COVID-19 pandemic (2020–22) versus the pre-pandemic period (2015–19).

**Methods:**

We conducted a retrospective population-based time series study using nationwide surveillance system data on reported pneumonia cases, hospitalizations, and deaths in Peru from 2015 to 2022. Age- and region-specific incidence rates (IRs) and incidence rate ratios (IRRs) with 95% confidence intervals were estimated for pneumonia cases, hospitalizations, and deaths by comparing the pandemic period (2020–22) with the pooled pre-pandemic period (2015–19).

**Results:**

Between January 1, 2015, and December 31, 2022, 170,558 pneumonia cases were reported among children younger than 5 years and 163,938 among adults aged >60 years in Peru. During the same period, 56,680 and 68,476 hospitalizations and 1,563 and 16,485 deaths occurred in these age groups, respectively. Compared with the pre-pandemic period (2015–2019), pneumonia incidence among children younger than 5 years declined markedly during the pandemic, decreasing by 71% in 2020 (IRR, 0.29; 95% CI, 0.28–0.30), 63% in 2021 (IRR, 0.37; 95% CI, 0.36–0.38), and 12% in 2022 (IRR, 0.88; 95% CI, 0.87–0.90). Hospitalizations and mortality in this group also decreased. In contrast, adults aged >60 years experienced increased hospitalization and mortality rates, particularly in 2021 (hospitalizations: IRR, 2.04; 95% CI, 1.97–2.09; mortality: IRR, 4.28; 95% CI, 4.01–4.57). Substantial regional heterogeneity was observed across Peru.

**Conclusions:**

During the COVID-19 pandemic, pneumonia epidemiology in Peru showed contrasting patterns by age, with sustained declines in incidence, hospitalizations, and mortality among children younger than 5 years, but increased hospitalizations and mortality among adults aged >60 years, underscoring the need to strengthen surveillance, access to timely care, and vaccination programs.

## 1. Introduction

Despite advances in medical science, pneumonia remains a major cause of global morbidity and mortality, particularly in low- and middle-income countries (LMICs), where access to timely diagnosis and appropriate care is often limited [1]. Pneumonia continues to drive high rates of hospitalization among children under five years of age and is increasingly recognized as a leading cause of severe illness, hospital admission, and mortality among older adults. In 2023, pneumonia was responsible for an estimated 2.5 million deaths worldwide, representing a notable increase compared with 2021. Nearly one quarter of these deaths (approximately 600,000) occurred among children under five years, while almost half were among adults aged 70 years and older (around 1.2 million deaths). Together, these two age groups account for nearly 70% of global pneumonia-related mortality, highlighting their persistent vulnerability and the urgent need for sustained surveillance and targeted public health interventions [2,3].

Pneumonia remains a major public health problem in Peru, with a substantial burden concentrated among young children and older adults. Between 2003 and 2017, pneumonia accounted for 166,844 deaths nationwide, of which 6.4% (10,757 deaths) occurred among children under five years of age and 75.6% (126,215 deaths) among adults aged 65 years and older [4,5]. Across all age groups, more than 94% of pneumonia-related deaths were classified as unspecified, highlighting persistent diagnostic limitations [4,5]. Among infants, evidence from 2006 to 2021 indicates that pneumonia represented 65,565 outpatient visits (0.8% of respiratory consultations), 51,618 hospitalizations (51.5% of respiratory admissions), and 3,770 deaths (71.6% of respiratory-related mortality), underscoring the disproportionate severity of pneumonia in this vulnerable population [6].

Several population-based studies comparing pre-pandemic and pandemic periods have reported substantial reductions in the incidence and hospitalization rates of non–COVID-19 pneumonia during 2020–2021. For example, a population-based study conducted in France showed that, prior to the COVID-19 pandemic, the incidence of pneumonia-related consultations ranged from 10 cases per 100,000 population during spring and summer to 47 cases per 100,000 population during autumn and winter, with an average seasonal peak of 39 cases per 100,000 population; during the pandemic period (2020–2022), consultation rates declined markedly, remaining below 10 cases per 100,000 population in spring and summer and below 20 cases per 100,000 population in autumn and winter [7]. Compared with pre-pandemic baselines, hospital admissions declined markedly across multiple settings [8–12]. These reductions were particularly pronounced among children and older adults, with pneumonia-related healthcare visits and hospitalizations declining by up to 60–70% (incidence rate ratios approximately 0.3–0.4) during the first year of the pandemic [8,13], likely reflecting marked reductions in community transmission of common respiratory pathogens following the widespread implementation of non-pharmaceutical interventions, including mask use, physical distancing, and school closures. In contrast, trends in pneumonia-related mortality were more heterogeneous. While some studies reported stable or modest declines in the absolute number of deaths and in-hospital mortality [8,11,14], several hospital-based and national analyses documented increased case-fatality rates and in-hospital mortality during the pandemic, particularly among older adults [9,10]. Relative increases in pneumonia mortality of approximately 7.5–12.1% compared with pre-pandemic periods have been reported [10], along with higher proportions of severe cases requiring intensive care and higher in-hospital mortality, which increased from 18.1% to 30.2% during the pandemic period [8]. These patterns likely reflect delayed healthcare-seeking behavior, reduced access to routine care, disruptions in diagnostic practices, and higher thresholds for hospital admission during periods of health system strain. As public health restrictions were progressively relaxed, several studies observed a rebound in pneumonia incidence rates among children [13] and hospitalization rates among adults [9], in some settings returning to or exceeding pre-pandemic levels, particularly in pediatric populations [13]. Overall, the available evidence indicates that the COVID-19 pandemic temporarily reshaped pneumonia epidemiology in complex and age-specific ways, characterized by short-term reductions in incidence and hospitalizations but potential increases in disease severity and mortality among high-risk groups [7–14].

Despite pneumonia remaining a major public health challenge in Peru, particularly among young children and older adults, comprehensive assessments quantifying changes in pneumonia incidence, hospitalizations, and mortality during the COVID-19 pandemic compared with the pre-pandemic period are lacking. Moreover, the true burden of pneumonia in these populations is likely underestimated as a result of disruptions in healthcare utilization, disease surveillance, and diagnostic practices during the pandemic. Addressing this evidence gap is essential to inform post-pandemic recovery strategies, strengthen respiratory disease surveillance systems, and support evidence-based policy decisions, including the evaluation and potential implementation of preventive interventions such as respiratory syncytial virus (RSV) vaccination in children and older adults.

Therefore, the objective of this study was to assess changes in the incidence, hospitalizations, and mortality rates due to pneumonia among children under five years of age and adults older than 60 years in Peru during the COVID-19 pandemic (2020–2022), compared with the pre-pandemic period (2015–2019), using national surveillance data.

## 2. Methods

### 2.1. Study design and data sources

We conducted a retrospective population-based time series study using data on reported pneumonia cases, hospitalizations, and deaths in Peru from January 1, 2015, to December 31, 2022, covering all 25 regions of the country. The study was conducted in accordance with the Strengthening the Reporting of Observational Studies in Epidemiology (STROBE) guidelines (**S1 Table**) [15]. Records of cases, hospitalizations, and deaths were obtained from the entire Peruvian health system, including the Ministry of Health (MINSA), Regional Health Directorates, Social Security (EsSalud), the Armed Forces and Police Health Services, and the private health sector.

Data were extracted from the Peruvian public health surveillance system dataset [16]. This dataset compiles information on the notification of pneumonia cases reported to the national public health surveillance system of Peru, which is administered by the National Center for Epidemiology, Prevention, and Disease Control (CDC Peru) of MINSA. The records originate from the National Epidemiology Network (RENACE), which comprises 10,232 health facilities from MINSA, EsSalud, and other institutions within the national health system. The dataset includes a historical time series of reported cases spanning from 2000 to 2022 [16].

A case of pneumonia was defined based on the clinical and syndromic records compiling information on the notification of acute respiratory infections. Due to the nature of these national registries, specific ICD-10 codes differentiating bacterial, viral (including SARS-CoV-2 infection), or unspecified pneumonia were not available, preventing the explicit exclusion of confirmed cases of COVID-19 from the aggregate counts. Consequently, the national system registers or records outcomes as events. Clinical pneumonia cases secondary to SARS-CoV-2 during the pandemic period (2020–2022) were indistinguishable from non-COVID-19 pneumonia within the national public health dataset.

The collected data included the department (geographical region), year and epidemiological week of case notification; non-pneumonia acute respiratory infection (ARI) cases in children under 5 years; pneumonia cases in children under 5 years; and pneumonia cases in adults older than 60 years. We also included pneumonia-related hospitalizations in children under 5 years (including children aged 0–4 completed years) and adults older than 60 years (including adults aged 61 years and older), as well as pneumonia-related deaths in both age groups. Mortality data for respiratory infections were obtained from the National Death Registry System (Sistema Nacional de Defunciones, SINADEF) of MINSA. Pneumonia-related mortality data extracted from SINADEF are classified based on the underlying cause of death. No additional information was available on the website of the CDC Peru.

To obtain the denominators for the rate calculations, annual regional population estimates and projections for children younger than 5 years and adults older than 60 years for the 2015–2022 period were obtained from the National Institute of Statistics and Informatics of Peru (INEI) [17].

### 2.2. Outcome

The primary outcomes evaluated in this study were age-specific pneumonia incidence, hospitalization, and mortality rates per 100,000 inhabitants. The parameters of interest were the relative changes in these measures between the pre-pandemic period (2015–19) and the pandemic period (2020–22).

### 2.3. Statistical analyses

We estimated changes in pneumonia case reporting, hospitalizations, and deaths during the COVID-19 pandemic (2020–2022) across geographical regions. The mean annual number of pneumonia cases, hospitalizations, and deaths during the pre-pandemic period (2015–2019) was used as the baseline for comparison. This five-year regional average served as the reference to assess variations observed during the COVID-19 pandemic years (2020–2022).

Annual crude incidence rates (IRs) for pneumonia cases, hospitalizations, and deaths presented in the tables were calculated per 10,000 children or older adults as the number of reported events divided by the corresponding annual population estimates (national or regional) and multiplied by 10,000. Weekly rates presented in the figures were calculated using the corresponding population denominators and expressed per 100,000 children or older adults. Age-specific (children aged <5 years and adults aged >60 years) and region-specific IRs for cases, hospitalizations, and deaths during the pre-pandemic period (2015–2019) were calculated by dividing the total number of pneumonia cases, hospitalizations, and deaths by the sum of the annual population estimates across the five-year period and expressed per 10,000 children or older adults. Annual population estimates by region used to calculate IRs for cases, hospitalizations, and deaths were obtained from population projections provided by the INEI of Peru [17]. Exact 95% confidence intervals (CIs) were calculated for all incidence rates (IRs) for cases, hospitalizations, and deaths assuming a Poisson distribution.

Exact 95% CIs for the estimated incidence rate ratios (IRRs), defined as incidence rate ratios for pneumonia cases, hospitalizations, and deaths, were calculated by comparing annual pneumonia IRs for cases, hospitalizations, and deaths during the COVID-19 pandemic period (2020–2022) with the pooled pre-pandemic IRs (2015–2019), which served as the reference period.

Adjusted incidence rate ratios (aIRRs) for pneumonia incidence, hospitalization, and mortality were estimated using generalized linear Poisson regression models with a log link. For analyses of incidence, hospitalization, and mortality, the annual numbers of pneumonia cases, hospitalizations, and deaths were used as the dependent variables, respectively, with the logarithm of the corresponding population estimate included as an offset to account for differences in population size. To evaluate mortality among reported pneumonia cases and mortality among hospitalized pneumonia patients, additional Poisson regression models were fitted using the annual number of pneumonia deaths as the dependent variable, with the logarithm of the annual number of reported pneumonia cases or hospitalized pneumonia cases included as the offset, respectively. The pre-pandemic period (2015–2019) was used as the reference category, and indicator variables were included for each study year (2020, 2021, and 2022). All models were adjusted for epidemiological week of case notification and geographic region. IRRs and aIRRs, with their corresponding 95% CIs, were estimated. A two-sided p value <0.05 was considered statistically significant.

All statistical analyses were performed using Stata/SE v17.0 (StataCorp, College Station, TX, USA).

### 2.4. Ethics

This study used anonymized, publicly available routine surveillance data from the national health system. Under local regulations, formal ethics review is waived for analyses of such public, non-identifiable data. As no identifiable information or direct human participation was involved, institutional ethics committee approval was not required. Informed consent was not required due to the observational nature of the study. Furthermore, all data sets were aggregated data and therefore completely anonymous.

## 3. Results

From January 1, 2015, to December 31, 2022, a total of 170 558 pneumonia cases were reported among children younger than 5 years and 163 938 cases among adults aged >60 years in Peru. During the same period, 56 680 hospitalizations for pneumonia occurred among children younger than 5 years and 68 476 among adults aged >60 years. In addition, 1 563 deaths due to pneumonia were recorded among children younger than 5 years and 16 485 deaths among adults aged >60 years.

### 3.1. Weekly pneumonia incidence, hospitalization, and mortality rates

Compared with the pre-pandemic period (2015–2019), weekly pneumonia indicators changed markedly during 2020–2022 (Fig 1A**-**1F). In 2020, weekly incidence declined sharply in children aged <5 years to <5 per 100,000 children younger than 5 years (Fig 1A), while adults aged >60 years showed moderate variability (Fig 1B). In 2021, incidence remained low in children but increased substantially in adults, peaking at 30 per 100,000 adults older than 60 years (Fig 1B), exceeding pre-pandemic levels. Weekly hospitalization rates decreased in children throughout 2020–2021 (<3 per 100,000 children younger than 5 years; Fig 1C) but increased in adults, particularly in 2021 (15–18 per 100,000 adults older than 60 years; Fig 1D). Weekly mortality remained low in children (<0.5 per 100,000 children younger than 5 years; Fig 1E) but rose markedly in adults in 2021, reaching 8–10 per 100,000 adults older than 60 years (Fig 1F). In 2022, all indicators partially rebounded toward pre-pandemic patterns.

**Fig 1:**
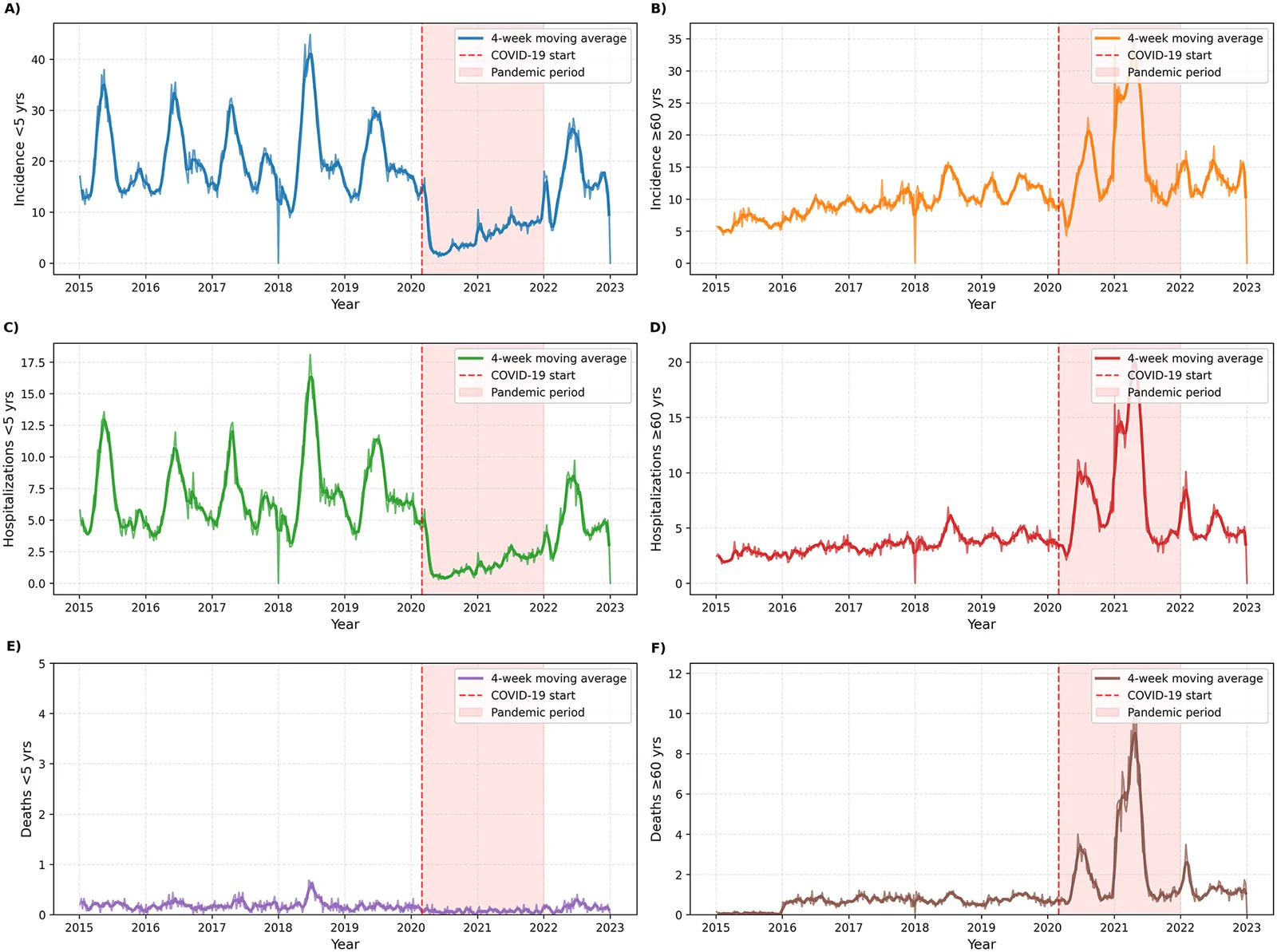
Weekly time series of six key pneumonia indicators in Peru, 2015–2022. Incidence rates for children aged <5 years **(A)** and adults aged >60 years **(B)**, expressed per 100,000 children or older adults. Hospitalization rates for children aged <5 years **(C)** and adults aged >60 years **(D)**, expressed per 100,000 children or older adults. Mortality rates for children aged <5 years **(E)** and adults aged >60 years **(F)**, expressed per 100,000 children or older adults. The plots show both raw weekly data and 4-week moving averages. Vertical lines indicate the onset of the COVID-19 pandemic (March 2020). Shaded areas represent the pandemic (2020–2022) period, highlighting temporal patterns such as seasonal peaks and potential disruptions associated with the COVID-19 period, and providing a comprehensive national overview of pneumonia epidemiology over the study period.

### 3.2. Changes in annual incidence, hospitalization, and mortality rates compared with the pre-pandemic period

Compared with the pre-pandemic period (2015–2019), pneumonia incidence among children younger than 5 years was 71% lower in 2020 (IRR, 0.29; 95% CI, 0.28–0.30), 63% lower in 2021 (IRR, 0.37; 95% CI, 0.36–0.38), and 12% lower in 2022 (IRR, 0.88; 95% CI, 0.87–0.90). Among adults aged >60 years, incidence increased by 3% in 2020 (IRR, 1.03; 95% CI, 1.01–1.05) and 2022 (IRR, 1.03; 95% CI, 1.01–1.05), and showed a marked increase of 51% in 2021 (IRR, 1.51; 95% CI, 1.48–1.54) (Table 1).

**Table 1:** Pneumonia incidence, hospitalization, and mortality rates in children aged <5 years and adults aged >60 years in Peru.

	Pre-pandemic (2015–19)		2020			2021			2022		
	n (mean)	IR (95% CI)	n	IR (95% CI)	IRR (95% CI)	n	IR (95% CI)	IRR (95% CI)	n	IR (95% CI)	IRR (95% CI)
**Children <5 years**											
Incidence	26366	93.11 (91.98 - 94.24)	7520	26.97 (26.36 - 27.58)	0.29 (0.28 - 0.30)	9396	34.66 (33.96 - 35.37)	0.37 (0.36 - 0.38)	21810	82.42 (81.33 - 83.53)	0.88 (0.87 - 0.90)
Hospitalization	9066	32.01 (31.36 - 32.68)	2427	8.70 (8.36 - 9.06)	0.27 (0.26 - 0.28)	2475	9.13 (8.77 - 9.50)	0.28 (0.27 - 0.30)	6449	24.37 (23.78 - 24.97)	0.76 (0.74 - 0.79)
Mortality	238	0.84 (0.74 - 0.95)	97	0.35 (0.28 - 0.42)	0.41 (0.32 - 0.53)	93	0.34 (0.28 - 0.42)	0.41 (0.32 - 0.52)	193	0.73 (0.63 - 0.84)	0.87 (0.71 - 1.05)
**Older adults >60 years**											
Incidence	16871	52.16 (51.37 - 52.95)	22343	53.96 (53.26 - 54.67)	1.03 (1.01 - 1.05)	33533	78.56 (77.72 - 79.41)	1.51 (1.48 - 1.54)	23705	53.71 (53.03 - 54.39)	1.03 (1.01 - 1.05)
Hospitalization	6241	19.29 (18.82 - 19.78)	11088	26.78 (26.28 - 27.28)	1.39 (1.34 - 1.43)	16783	39.32 (38.73 - 39.92)	2.04 (1.97 - 2.09)	9400	21.29 (20.87 - 21.73)	1.10 (1.07 - 1.14)
Mortality	1091	3.37 (3.18 - 3.58)	2532	6.11 (5.88 - 6.36)	1.81 (1.68 - 1.95)	6161	14.43 (14.08 - 14.80)	4.28 (4.01 - 4.57)	2338	5.30 (5.08 - 5.52)	1.57 (1.46 - 1.69)

Abbreviations: IR, incidence rate per 10,000 children or adults; IRR, incidence rate ratio; CI, confidence interval.

Hospitalization rates for pneumonia among children younger than 5 years were 73% lower in 2020 (IRR, 0.27; 95% CI, 0.26–0.28), 72% lower in 2021 (IRR, 0.28; 95% CI, 0.27–0.30), and 24% lower in 2022 (IRR, 0.76; 95% CI, 0.74–0.79) compared with the pre-pandemic period. In contrast, hospitalization rates among adults aged >60 years were 39% higher in 2020 (IRR, 1.39; 95% CI, 1.34–1.43), 104% higher in 2021 (IRR, 2.04; 95% CI, 1.97–2.09), and 10% higher in 2022 (IRR, 1.10; 95% CI, 1.07–1.14) (Table 1).

Death rates from pneumonia among children younger than 5 years were 59% lower in 2020 and 2021 (IRR, 0.41; 95% CI, 0.32–0.53 and 0.32–0.52, respectively) and 13% lower in 2022 (IRR, 0.87; 95% CI, 0.71–1.05) compared with the pre-pandemic period. Among adults aged >60 years, death rates were 81% higher in 2020 (IRR, 1.81; 95% CI, 1.68–1.95), 328% higher in 2021 (IRR, 4.28; 95% CI, 4.01–4.57), and 57% higher in 2022 (IRR, 1.57; 95% CI, 1.46–1.69) (Table 1).

### 3.3. Adjusted incidence rate ratios for pneumonia incidence, hospitalization, and mortality

Among children aged <5 years, compared with the pre-pandemic period, adjusted pneumonia incidence was 59% lower in 2020 (adjusted incidence rate ratio [aIRR], 0.41; 95% CI, 0.39–0.43; *p* < 0.001), 54% lower in 2021 (aIRR, 0.46; 95% CI, 0.44–0.48; *p* < 0.001), and 6% lower in 2022 (aIRR, 0.94; 95% CI, 0.90–0.97; *p* = 0.001). Hospitalization rates were 62% lower in 2020 (aIRR, 0.38, 95% CI 0.36–0.41; *p* < 0.001), 65% lower in 2021 (aIRR, 0.35, 95% CI 0.33–0.37; *p* < 0.001), and 19% lower in 2022 (aIRR, 0.81, 95% CI 0.77–0.84; *p* < 0.001). Mortality was also significantly reduced, by 41% in 2020 (aIRR, 0.59, 95% CI 0.47–0.74; *p* < 0.001) and 50% in 2021 (aIRR, 0.50, 95% CI 0.41–0.62; *p* < 0.001) (Table 2).

**Table 2:** Adjusted incidence rate ratios estimated using generalized linear Poisson models for incidence, hospitalizations, and mortality due to pneumonia, Peru.

	Incidence				Hospitalization				Mortality			
	IRR (95% CI)	p value	aIRR (95% CI)	p value	IRR (95% CI)	p value	aIRR (95% CI)	p value	IRR (95% CI)	p value	aIRR (95% CI)	p value
**Children aged <5 years**												
Pre-pandemic (2015–19)	1.00 (Ref.)		1.00 (Ref.)		1.00 (Ref.)		1.00 (Ref.)		1.00 (Ref.)		1.00 (Ref.)	
2020	0.41 (0.39 - 0.43)	<0.001	0.41 (0.39 - 0.43)	<0.001	0.38 (0.36 - 0.41)	<0.001	0.38 (0.36 - 0.41)	<0.001	0.58 (0.46 - 0.73)	<0.001	0.59 (0.47 - 0.74)	<0.001
2021	0.46 (0.44 - 0.48)	<0.001	0.46 (0.44 - 0.48)	<0.001	0.35 (0.33 - 0.37)	<0.001	0.35 (0.33 - 0.37)	<0.001	0.50 (0.41 - 0.62)	<0.001	0.50 (0.41 - 0.62)	<0.001
2022	0.93 (0.90 - 0.97)	0.001	0.94 (0.90 - 0.97)	0.001	0.81 (0.77 - 0.84)	<0.001	0.81 (0.77 - 0.84)	<0.001	0.92 (0.79 −1.08)	0.301	0.92 (0.79 - 1.07)	0.297
**Adults aged >60 years**												
Pre-pandemic (2015–19)	1.00 (Ref.)		1.00 (Ref.)		1.00 (Ref.)		1.00 (Ref.)		1.00 (Ref.)		1.00 (Ref.)	
2020	1.37 (1.30 - 1.44)	<0.001	1.37 (1.31 - 1.44)	<0.001	1.84 (1.71 - 1.97)	<0.001	1.84 (1.71 - 1.97)	<0.001	2.40 (2.19 - 2.63)	<0.001	2.35 (2.14 - 2.57)	<0.001
2021	1.79 (1.71 - 1.88)	<0.001	1.81 (1.72 - 1.89)	<0.001	2.43 (2.24 - 2.62)	<0.001	2.45 (2.28 - 2.64)	<0.001	5.09 (4.49 - 5.78)	<0.001	5.12 (4.52 - 5.79)	<0.001
2022	1.03 (0.99 - 1.07)	0.114	1.04 (1.00 - 1.08)	0.033	1.10 (1.05 - 1.16)	<0.001	1.12 (1.07 - 1.18)	<0.001	1.57 (1.45 - 1.70)	<0.001	1.59 (1.46 - 1.72)	<0.001

aIRR: adjusted for epidemiological week of notification and geographic region using generalized linear Poisson regression models with the logarithm of the corresponding population as an offset.

Abbreviations: IRR, incidence rate ratio; aIRR, adjusted incidence rate ratio.

Among adults aged >60 years, compared with the pre-pandemic period, adjusted pneumonia incidence was 37% higher in 2020 (aIRR, 1.37; 95% CI, 1.31–1.44; *p* < 0.001), 81% higher in 2021 (aIRR, 1.81; 95% CI, 1.72–1.89; *p* < 0.001), and 4% higher in 2022 (aIRR, 1.04; 95% CI, 1.00–1.08; *p* = 0.033). Hospitalization rates were 84% higher in 2020 (aIRR, 1.84; 95% CI, 1.71–1.97; *p* < 0.001), 145% higher in 2021 (aIRR, 2.45; 95% CI, 2.28–2.64; *p* < 0.001), and 12% higher in 2022 (aIRR, 1.12; 95% CI, 1.07–1.18; *p* < 0.001). Mortality increased by 135% in 2020 (aIRR, 2.35; 95% CI, 2.14–2.57; *p* < 0.001), 412% in 2021 (aIRR, 5.12; 95% CI, 4.52–5.79; *p* < 0.001), and 59% in 2022 (aIRR, 1.59; 95% CI, 1.46–1.72; *p* < 0.001) (Table 2).

### 3.4. Adjusted mortality among reported pneumonia cases and hospitalized pneumonia patients

Among children aged <5 years, mortality among reported pneumonia cases was 43% higher in 2020 (aIRR, 1.43; 95% CI, 1.13–1.79; *p* = 0.003) than during the pre-pandemic period, but returned to levels comparable to the pre-pandemic period in 2021 and 2022. Mortality among hospitalized pneumonia patients was 62% higher in 2020 (aIRR, 1.62; 95% CI, 1.23–2.14; *p* = 0.001) and remained 38% higher in 2021 (aIRR, 1.38; 95% CI, 1.06–1.80; *p* = 0.016), before returning to pre-pandemic levels in 2022 (Table 3).

**Table 3:** Adjusted incidence rate ratios for mortality among reported pneumonia cases and hospitalized pneumonia patients, Peru.

	Mortality among reported pneumonia cases				Mortality among hospitalized pneumonia patients			
	IRR (95% CI)	p value	aIRR (95% CI)	p value	IRR (95% CI)	p value	aIRR (95% CI)	p value
**Children aged <5 years**								
Pre-pandemic (2015–19)	1.00 (Ref.)		1.00 (Ref.)		1.00 (Ref.)		1.00 (Ref.)	
2020	1.43 (1.14 - 1.79)	0.002	1.43 (1.13 - 1.79)	0.003	1.68 (1.29 - 2.20)	<0.001	1.62 (1.23 - 2.14)	0.001
2021	1.09 (0.89 - 1.35)	0.394	1.10 (0.89 - 1.36)	0.389	1.41 (1.09 - 1.83)	0.010	1.38 (1.06 - 1.80)	0.016
2022	0.98 (0.84 - 1.14)	0.803	0.98 (0.84 - 1.15)	0.808	1.12 (0.92 - 1.36)	0.242	1.11 (0.92 - 1.35)	0.271
**Adults aged >60 years**								
Pre-pandemic (2015–19)	1.00 (Ref.)		1.00 (Ref.)		1.00 (Ref.)		1.00 (Ref.)	
2020	1.74 (1.59 - 1.90)	<0.001	1.77 (1.62 - 1.93)	<0.001	1.40 (1.27 - 1.55)	<0.001	1.39 (1.25 - 1.52)	<0.001
2021	2.84 (2.57 - 3.14)	<0.001	2.67 (2.43 - 2.92)	<0.001	2.33 (2.16 - 2.52)	<0.001	2.16 (2.02 - 2.31)	<0.001
2022	1.53 (1.42 - 1.64)	<0.001	1.51 (1.41 - 1.62)	<0.001	1.52 (1.42 - 1.63)	<0.001	1.49 (1.39 - 1.59)	<0.001

Adjusted incidence rate ratio (aIRR), adjusted for epidemiological week of notification and geographic region.

Among adults aged >60 years, compared with the pre-pandemic period, mortality among reported pneumonia cases was 77% higher in 2020 (aIRR, 1.77; 95% CI, 1.62–1.93; *p* < 0.001), increased to 167% higher in 2021 (aIRR, 2.67; 95% CI, 2.43–2.92; *p* < 0.001), and remained 51% higher in 2022 (aIRR, 1.51; 95% CI, 1.41–1.62; *p* < 0.001). Similarly, mortality among hospitalized pneumonia patients was 39% higher in 2020 (aIRR, 1.39; 95% CI, 1.25–1.52; *p* < 0.001), increased to 116% higher in 2021 (aIRR, 2.16; 95% CI, 2.02–2.31; *p* < 0.001), and remained 49% higher in 2022 (aIRR, 1.49; 95% CI, 1.39–1.59; *p* < 0.001) (Table 3).

### 3.5. Pneumonia incidence by region

Compared with the pre-pandemic period (2015–2019), pneumonia incidence rates declined markedly across most regions in 2020 in both children aged <5 years and adults aged >60 years (Fig 2A, 2B). In children, several regions decreased from high pre-pandemic levels (120–180 per 100,000 children younger than 5 years) to below 60 in 2020, with partial increases in 2021 and a clearer rebound in 2022, when some regions again exceeded 140 per 100,000 children younger than 5 years (Fig 2A). In adults, incidence remained more stable in 2020 (generally 40–100 per 100,000 adults older than 60 years) but increased in 2021, with some regions reaching >200–300 per 100,000 adults older than 60 years, before declining to intermediate levels in 2022 (Fig 2B). Overall, 2022 showed a partial return toward pre-pandemic geographic patterns, particularly in children.

**Fig 2:**
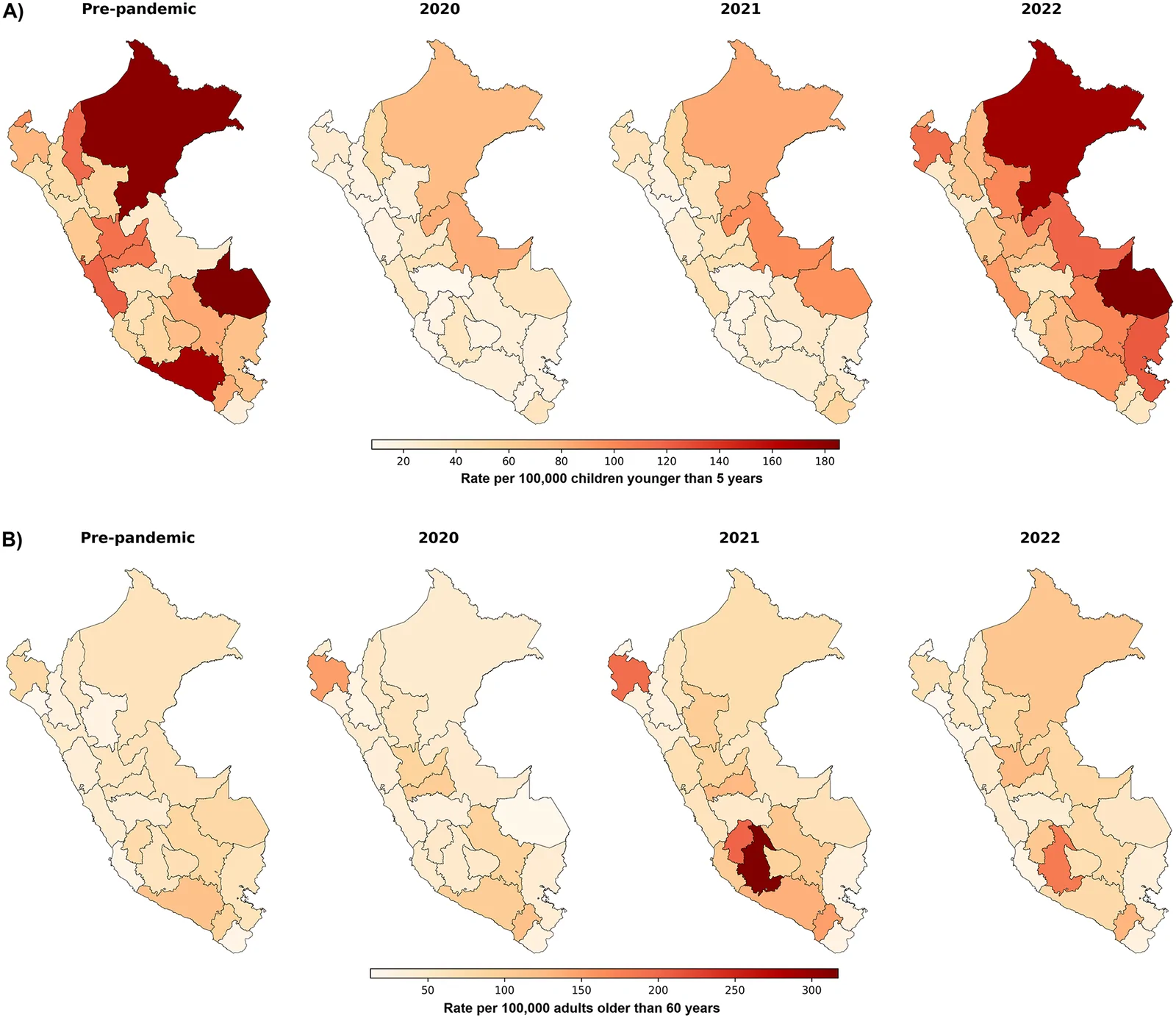
Pneumonia incidence rates in children aged <5 years (A) and adults aged >60 years (B) before and during the COVID-19 pandemic in Peru.

Among children aged <5 years, the marked reductions in 2020 were observed in Cajamarca (IRR 0.18, 95% CI 0.16–0.21), Huancavelica (IRR 0.17, 95% CI 0.13–0.23), and Cusco (IRR 0.24, 95% CI 0.21–0.27). Similar decreases persisted in 2021 across many regions, including Callao (IRR, 0.23; 95% CI, 0.19–0.26) and Lima (IRR, 0.45; 95% CI, 0.43–0.46). In 2022, incidence partially rebounded but remained below pre-pandemic levels in most regions; however, increases above baseline were observed in Madre de Dios (IRR, 1.97; 95% CI, 1.77–2.20), Loreto (IRR, 1.84; 95% CI, 1.76–1.92), Puno (IRR, 1.32; 95% CI, 1.24–1.41), and Ucayali (IRR, 1.27; 95% CI, 1.19–1.36) (Table 4).

**Table 4:** Pneumonia incidence rates among children aged <5 years and adults aged >60 years, by region (IR, incidence rate per 10,000 children or adults; IRR, incidence rate ratio; CI, confidence interval).

	Incidence rate in children <5 years					Incidence rate in adults >60 years				
	Pre-pandemic (2015–19)		2020	2021	2022	Pre-pandemic (2015–19)		2020	2021	2022
	n (mean)	IR (95% CI)	IRR (95% CI)	IRR (95% CI)	IRR (95% CI)	n (mean)	IR (95% CI)	IRR (95% CI)	IRR (95% CI)	IRR (95% CI)
Amazonas	495	116.58 (106.53 - 127.32)	0.50 (0.44 - 0.57)	0.54 (0.47 - 0.61)	0.79 (0.72 - 0.88)	188	53.32 (45.97 - 61.51)	1.01 (0.82 - 1.25)	1.13 (0.95 - 1.38)	0.88 (0.72 - 1.09)
Ancash	667	62.26 (57.62 - 67.16)	0.18 (0.15 - 0.21)	0.26 (0.23 - 0.30)	0.68 (0.62 - 0.73)	472	38.29 (34.92 - 41.91)	0.92 (0.81 - 1.04)	1.84 (1.65 - 2.06)	1.32 (1.17 - 1.48)
Apurimac	350	71.79 (64.46 - 79.72)	0.17 (0.13 - 0.22)	0.20 (0.16 - 0.25)	0.77 (0.68 - 0.87)	284	67.67 (60.02 - 76.02)	0.76 (0.63 - 0.91)	1.35 (1.16 - 1.58)	1.02 (0.86 - 1.20)
Arequipa	1753	168.89 (161.08 - 176.99)	0.22 (0.19 - 0.24)	0.28 (0.25 - 0.32)	1.03 (0.96 - 1.09)	1805	116.90 (111.57- 122.42)	0.86 (0.80 - 0.91)	1.16 (1.09 - 1.22)	0.68 (0.64 - 0.72)
Ayacucho	398	52.15 (47.15 −57.54)	0.12 (0.10 - 0.15)	0.29 (0.25 - 0.34)	0.82 (0.74 - 0.90)	339	60.10 (53.87 - 66.85)	0.78 (0.66 - 0.92)	5.27 (4.70 - 5.94)	3.02 (2.67 - 3.42)
Cajamarca	719	48.98 (45.47 - 52.70)	0.18 (0.16 - 0.21)	0.20 (0.18 - 0.23)	0.72 (0.67 - 0.77)	399	29.77 (26.92 - 32.84)	0.89 (0.77 - 1.03)	1.06 (0.92 - 1.21)	1.99 (1.78 - 2.25)
Callao	883	113.31 (105.96 - 121.04)	0.27 (0.24 - 0.31)	0.23 (0.19 - 0.26)	0.65 (0.59 - 0.71)	892	73.54 (68.79 - 78.53)	1.07 (0.98 - 1.16)	1.03 (0.95 −1.12)	0.66 (0.60 - 0.72)
Cusco	1009	83.41 (78.35 - 88.72)	0.24 (0.21 - 0.27)	0.33 (0.30 - 0.37)	1.09 (1.03 - 1.16)	1151	85.68 (80.79 - 90.77)	1.09 (1.01 - 1.18)	1.29 (1.20 - 1.40)	0.94 (0.87 - 1.02)
Huancavelica	268	41.19 (36.41 - 46.44)	0.17 (0.13 - 0.21)	0.17 (0.13 - 0.23)	0.56 (0.48 - 0.64)	266	77.48 (68.45 - 87.38)	0.67 (0.55 - 0.82)	2.60 (2.25 - 3.01)	1.40 (1.19 - 1.66)
Huanuco	1022	112.00 (105.24 - 119.08)	0.33 (0.29 - 0.38)	0.40 (0.36 - 0.45)	0.88 (0.81 - 0.95)	453	59.86 (54.47 - 65.64)	1.50 (1.32 - 1.70)	1.56 (1.38 - 1.77)	2.12 (1.89 - 2.39)
Ica	344	51.66 (46.35 - 57.42)	0.12 (0.10 - 0.15)	0.14 (0.12 - 0.17)	0.12 (0.10 - 0.15)	207	23.30 (20.23 - 26.69)	2.06 (1.76 - 2.43)	4.44 (3.83 - 5.18)	1.14 (0.96 - 1.37)
Junin	550	38.90 (35.72 - 42.29)	0.11 (0.09 - 0.13)	0.15 (0.13 - 0.17)	0.39 (0.36 - 0.43)	519	40.77 (37.34 - 44.43)	0.73 (0.64 - 0.82)	1.24 (1.11 - 1.39)	0.96 (0.86 - 1.08)
La Libertad	667	39.45 (36.51 - 42.56)	0.14 (0.12 - 0.16)	0.09 (0.07 - 0.10)	0.29 (0.26 - 0.32)	945	48.33 (45.30 - 51.51)	0.97 (0.89 - 1.06)	0.87 (0.80 - 0.95)	0.47 (0.42 - 0.52)
Lambayeque	477	44.79 (40.86 - 49.00)	0.22 (0.19 - 0.25)	0.28 (0.25 - 0.31)	0.34 (0.31 - 0.38)	218	15.14 (13.19 - 17.28)	2.01 (1.72 - 2.37)	2.20 (1.88 - 2.58)	0.83 (0.69 - 1.01)
Lima	9676	120.36 (117.97 - 122.78)	0.36 (0.34 - 0.37)	0.45 (0.43 - 0.46)	0.97 (0.95 - 1.00)	4936	42.38 (41.20 - 43.58)	0.91 (0.88 - 0.95)	1.45 (1.40 - 1.50)	1.07 (1.04 - 1.11)
Loreto	1965	180.35 (172.46 - 188.51)	0.79 (0.74 - 0.84)	0.89 (0.84 - 0.95)	1.84 (1.76 - 1.92)	460	60.63 (55.22 - 66.43)	0.73 (0.63 - 0.84)	1.20 (1.06 - 1.35)	1.81 (1.61 - 2.02)
Madre de Dios	239	185.47 (162.70 - 210.54)	0.39 (0.29 - 0.51)	1.02 (0.87 - 1.18)	1.97 (1.77 - 2.20)	70	82.24 (64.11 - 103.90)	0.17 (0.10 - 0.28)	0.80 (0.59 - 1.09)	0.66 (0.48 - 0.91)
Moquegua	110	82.77 (68.03 - 99.77)	0.14 (0.08 - 0.22)	0.04 (0.01 - 0.10)	0.45 (0.33 - 0.59)	201	92.08 (79.79 - 105.73)	1.26 (1.06 - 1.51)	1.62 (1.37 - 1.93)	1.42 (1.20 - 1.70)
Pasco	338	109.19 (97.86 - 121.48)	0.43 (0.35 - 0.53)	0.46 (0.37 - 0.56)	0.76 (0.65 - 0.89)	140	60.86 (51.20 - 71.82)	1.67 (1.35 - 2.06)	2.12 (1.73 - 2.59)	1.04 (0.83 - 1.30)
Piura	1404	77.43 (73.43 −81.59)	0.29 (0.26 - 0.31)	0.41 (0.38 - 0.45)	1.21 (1.16 - 1.27)	1445	81.51 (77.36 - 85.82)	1.85 (1.74 - 1.96)	2.38 (2.25 - 2.53)	0.87 (0.82 - 0.94)
Puno	1006	69.66 (65.42 - 74.10)	0.19 (0.17 - 0.22)	0.26 (0.23 - 0.30)	1.32 (1.24 - 1.41)	859	62.72 (58.60 - 67.06)	0.64 (0.58 - 0.72)	0.50 (0.45 - 0.56)	0.61 (0.54 - 0.67)
San Martin	448	55.96 (50.89 - 61.39)	0.22 (0.19 - 0.25)	0.39 (0.35 - 0.44)	1.07 (0.99 - 1.14)	168	25.52 (21.81 - 29.69)	2.43 (2.03 - 2.91)	3.91 (3.31 - 4.64)	3.27 (2.76 - 3.88)
Tacna	60	21.47 (16.38 - 27.64)	0.04 (0.02 - 0.07)	0.06 (0.03 - 0.09)	0.36 (0.29 - 0.45)	69	21.07 (16.39 - 26.66)	1.38 (1.03 - 1.87)	1.18 (0.87 - 1.60)	1.35 (1.01 - 1.82)
Tumbes	192	96.75 (83.55 - 111.45)	0.18 (0.13 - 0.25)	0.42 (0.34 - 0.52)	0.90 (0.78 - 1.04)	112	57.19 (47.09 - 68.81)	0.76 (0.58 - 0.99)	0.41 (0.30 - 0.56)	0.31 (0.22 - 0.43)
Ucayali	1325	29.92 (28.33 - 31.58)	0.87 (0.80 - 0.95)	1.04 (0.97 - 1.13)	1.27 (1.19 - 1.36)	272	65.53 (57.97 - 73.80)	0.70 (0.58 - 0.83)	0.85 (0.72 - 1.00)	1.29 (1.11 - 1.50)
										

Among adults aged >60 years, pneumonia incidence increased in several regions during the pandemic, particularly in 2021. Notable increases were observed in Ayacucho (IRR, 5.27; 95% CI, 4.70–5.94), Ica (IRR, 4.44; 95% CI, 3.83–5.18), and San Martin (IRR, 3.91; 95% CI, 3.31–4.64). Elevated incidence also persisted in 2022 in some regions, including San Martín (IRR, 3.27; 95% CI, 2.76–3.88), Ayacucho (IRR, 3.02; 95% CI, 2.67–3.42) and Huánuco (IRR, 2.12; 95% CI, 1.89–2.39). However, decreases relative to the pre-pandemic period were observed in others, such as Tumbes (IRR, 0.31; 95% CI, 0.22–0.43), indicating substantial regional heterogeneity (Table 4).

### 3.6. Hospitalization rates by region

Pneumonia hospitalization rates declined markedly in children in 2020, whereas rates among adults showed substantial regional variation, with increases in several regions (Fig 3A, 3B). In children <5 years, high pre-pandemic levels dropped below 60 in 2020, followed by a significant rebound in 2022, when hospitalization rates increased in several regions and exceeded pre-pandemic levels in some regions (Fig 3A). Conversely, in adults aged >60 years, rates remained more stable in 2020 (40–100 per 100,000 adults older than 60 years) but surged in 2021, with some regions reaching >200–300 per 100,000 adults older than 60 years before declining to intermediate levels in 2022 (Fig 3B). Overall, 2022 showed a partial return toward pre-pandemic geographic patterns, particularly in children <5 years, while rates in adults aged >60 years remained elevated in specific regions compared to 2020.

**Fig 3:**
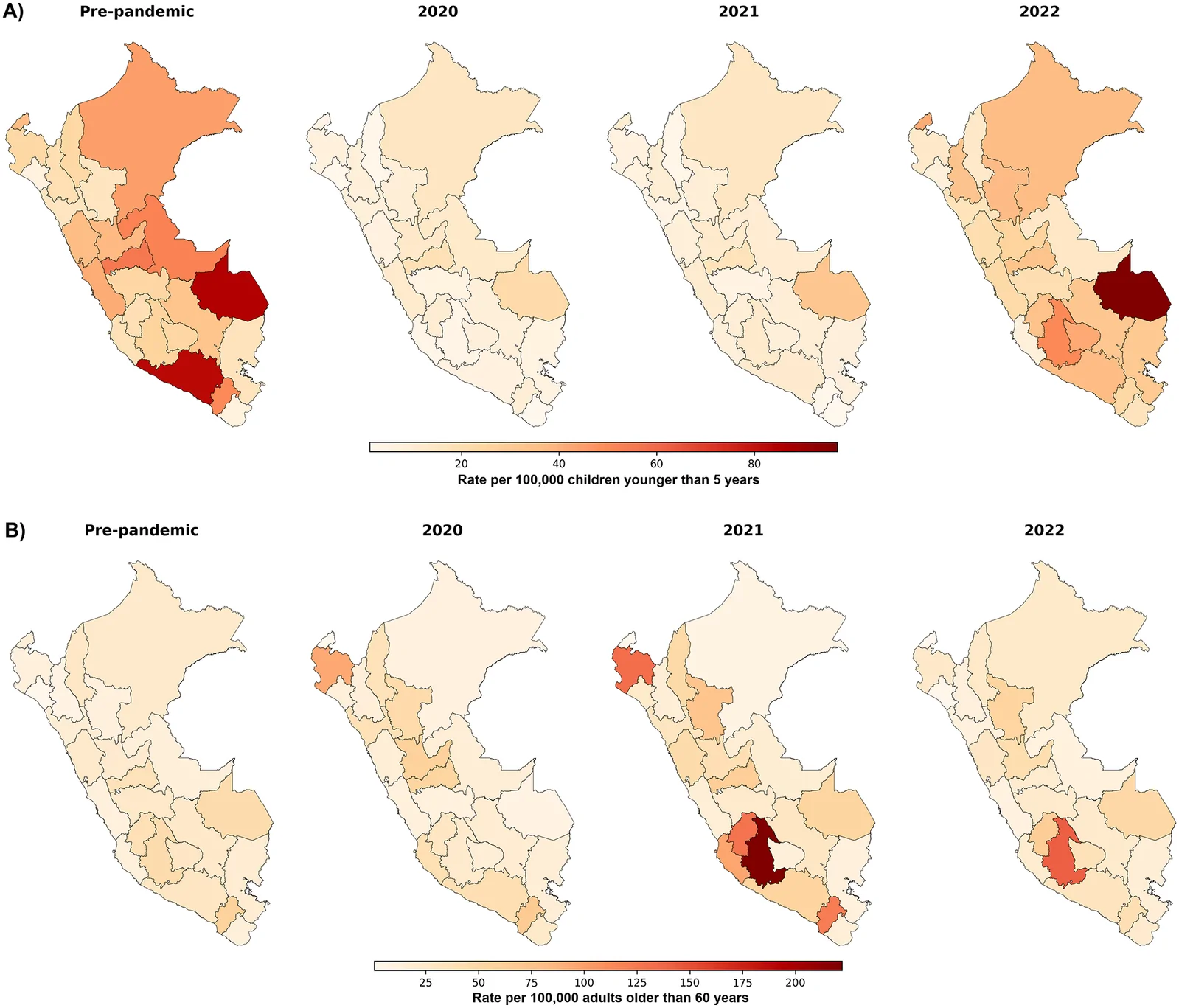
Pneumonia hospitalization rates in children aged <5 years (A) and adults aged >60 years (B) before and during the COVID-19 pandemic in Peru.

Hospitalization rates for pneumonia among children younger than 5 years declined substantially in most regions during 2020 and 2021. Marked reductions in 2020 were observed in Amazonas (IRR, 0.10; 95% CI, 0.05–0.19), Tumbes (IRR, 0.13; 95% CI, 0.10–0.23), and Ayacucho (IRR, 0.15; 95% CI, 0.09–0.22). Similar decreases persisted in 2021 across several areas, most notably in Moquegua (IRR, 0.05; 95% CI, 0.02–0.16) and Arequipa (IRR, 0.14; 95% CI, 0.12–0.18). In 2022, hospitalization rates remained below pre-pandemic levels in several regions, including Lima (IRR, 0.54; 95% CI, 0.51–0.58) and Piura (IRR, 0.55; 95% CI, 0.48–0.64), although increases above baseline were observed in Cajamarca (IRR, 1.73; 95% CI, 1.49–2.01) and San Martín (IRR, 2.39; 95% CI, 1.97–2.90) (Table 5).

**Table 5:** Pneumonia hospitalization rates among children aged <5 years and adults aged >60 years, by region (IR, incidence rate per 10,000 children or adults; IRR, incidence rate ratio; CI, confidence interval).

	Hopitalization rate in children <5 years					Hospitalization rate in adults >60 years				
	**Pre-pandemic (2015–19)**		**2020**	**2021**	**2022**	**Pre-pandemic (2015–19)**		**2020**	**2021**	**2022**
	**n (mean)**	**IR (95% CI)**	**IRR (95% CI)**	**IRR (95% CI)**	**IRR (95% CI)**	**n (mean)**	**IR (95% CI)**	**IRR (95% CI)**	**IRR (95% CI)**	**IRR (95% CI)**
Amazonas	100	23.55 (19.23-28.57)	0.10 (0.05 - 0.19)	0.14 (0.09 - 0.24)	0.55 (0.42 - 0.71)	106	30.06 (24.70 - 36.35)	1.27 (1.01 - 1.60)	1.65 (1.32 - 2.06)	0.94 (0.72 - 1.24)
Ancash	389	36.31 (32.83-40.07)	0.16 (0.12 - 0.21)	0.22 (0.17 - 0.28)	0.56 (0.48 - 0.65)	325	26.37 (23.58 - 29.42)	0.74 (0.65 - 0.84)	1.78 (1.63 - 1.94)	1.17 (1.02 - 1.35)
Apurimac	116	23.79 (19.78-28.38)	0.31 (0.20 - 0.46)	0.33 (0.22 - 0.51)	1.75 (1.41 - 2.17)	133	31.68 (26.63 - 37.42)	0.60 (0.45 - 0.80)	0.48 (0.37 - 0.64)	1.23 (0.98 - 1.55)
Arequipa	863	83.15 (77.78-88.84)	0.70 (0.05 - 0.90)	0.14 (0.12 - 0.18)	0.44 (0.39 - 0.49)	499	32.33 (29.57 - 35.30)	1.30 (1.18 - 1.42)	1.73 (1.60 - 1.87)	1.06 (0.94 - 1.18)
Ayacucho	193	25.28 (21.85-29.24)	0.15 (0.09 - 0.22)	0.48 (0.37 - 0.63)	1.99 (1.67 - 2.38)	260	46.10 (40.74 - 52.05)	0.54 (0.45 - 0.65)	2.43 (2.24 - 2.64)	3.90 (3.46 - 4.39)
Cajamarca	283	19.28 (17.07-21.79)	0.42 (0.33 - 0.52)	0.51 (0.41 - 0.63)	1.73 (1.49 - 2.01)	123	9.18 (7.61 - 11.04)	1.93 (1.57 - 2.37)	2.07 (1.79 - 2.39)	1.61 (1.37 - 1.90)
Callao	205	26.32 (22.86-30.21)	0.41 (0.32 - 0.52)	0.29 (0.22 - 0.39)	0.60 (0.48 - 0.75)	274	22.58 (19.97 - 25.42)	1.74 (1.53 - 1.98)	1.74 (1.59 - 1.91)	0.93 (0.83 - 1.04)
Cusco	397	32.82 (29.66-36.24)	0.25 (0.20 - 0.31)	0.28 (0.23 - 0.35)	0.97 (0.85 - 1.10)	375	27.91 (25.13 - 30.92)	0.78 (0.69 - 0.88)	1.14 (1.01 - 1.28)	0.89 (0.78 - 1.01)
Huancavelica	120	18.45 (15.35-22.17)	0.53 (0.36 - 0.77)	0.49 (0.34 - 0.73)	1.84 (1.45 - 2.35)	138	40.19 (33.85 - 47.26)	0.79 (0.59 - 1.05)	3.24 (2.79 - 3.77)	1.62 (1.36 - 1.92)
Huanuco	344	37.70 (33.85-41.93)	0.36 (0.28 - 0.45)	0.32 (0.25 - 0.40)	0.68 (0.58 - 0.81)	183	24.17 (20.83 - 28.02)	2.45 (2.08 - 2.89)	1.68 (1.41 - 2.01)	1.80 (1.50 - 2.15)
Ica	122	18.32 (15.26-21.99)	0.21 (0.14 - 0.31)	0.43 (0.33 - 0.57)	0.44 (0.32 - 0.61)	143	16.10 (13.62 - 19.03)	2.63 (2.26 - 3.05)	6.10 (5.31 - 7.02)	1.47 (1.23 - 1.75)
Junin	339	23.98 (21.51-26.7)	0.23 (0.18 - 0.30)	0.20 (0.15 - 0.26)	0.82 (0.70 - 0.97)	370	29.07 (26.17 - 32.26)	0.43 (0.37 - 0.52)	0.88 (0.77 - 1.02)	1.14 (1.01 - 1.29)
La Libertad	266	15.73 (13.9-17.78)	0.48 (0.43 - 0.57)	0.30 (0.24 - 0.38)	1.08 (0.94 - 1.25)	386	19.74 (17.78 - 21.87)	1.96 (1.77 - 2.17)	1.72 (1.59 - 1.87)	0.70 (0.62 - 0.80)
Lambayeque	66	6.20 (4.80-7.91)	0.32 (0.25 - 0.48)	1.01 (0.73 - 1.40)	1.30 (0.97 - 1.74)	39	2.71 (1.93 - 3.71)	5.83 (4.22 - 8.06)	10.02 (7.31 - 13.74)	1.32 (1.01 - 1.73)
Lima	3312	41.20 (39.8-42.64)	0.29 (0.28 - 0.31)	0.26 (0.24 - 0.28)	0.54 (0.51 - 0.58)	1641	14.09 (13.42 - 14.79)	0.96 (0.92 - 1.00)	1.33 (1.28 - 1.38)	0.84 (0.80 - 0.88)
Loreto	487	44.70 (40.89-48.84)	0.33 (0.31 - 0.38)	0.33 (0.28 - 0.39)	0.81 (0.71 - 0.93)	199	26.23 (22.73 - 30.17)	0.45 (0.35 - 0.59)	0.30 (0.24 - 0.38)	1.30 (1.10 - 1.54)
Madre de Dios	110	85.38 (70.09-103.42)	0.25 (0.21 - 0.36)	0.37 (0.27 - 0.52)	1.14 (0.93 - 1.40)	39	45.83 (32.60 - 62.77)	0.21 (0.11 - 0.40)	5.54 (4.07 - 7.55)	4.96 (3.64 - 6.76)
Moquegua	67	50.42 (39.08-64.02)	0.18 (0.14 - 0.32)	0.05 (0.02 - 0.16)	0.46 (0.30 - 0.72)	128	58.63 (48.76 - 69.97)	1.16 (0.96 - 1.41)	1.05 (0.92 - 1.20)	0.92 (0.75 - 1.13)
Pasco	171	55.25 (47.32-64.28)	0.33 (0.28 - 0.44)	0.31 (0.22 - 0.43)	0.61 (0.47 - 0.81)	88	38.26 (30.66 - 47.07)	1.42 (1.12 - 1.79	1.68 (1.42 - 1.98)	0.62 (0.44 - 0.87)
Piura	427	23.55 (21.38-25.91)	0.28 (0.25 - 0.33)	0.24 (0.20 - 0.29)	0.55 (0.48 - 0.64)	246	13.87 (12.18 - 15.76)	6.87 (6.13 - 7.70	9.63 (8.61 - 10.77)	2.03 (1.76 - 2.36)
Puno	241	16.68 (14.69-18.94)	0.34 (0.30 - 0.43)	0.43 (0.33 - 0.57)	1.83 (1.54 - 2.17)	301	21.98 (19.55 - 24.65)	0.80 (0.69 - 0.92	0.62 (0.54 - 0.72)	0.87 (0.75 - 1.01)
San Martin	124	15.48 (12.95-18.52)	0.60 (0.50 - 0.74)	0.59 (0.46 - 0.75)	2.39 (1.97 −2.90)	94	14.28 (11.57 - 17.43)	3.56 (2.82 - 4.49	6.44 (5.28 - 7.87)	5.19 (4.25 - 6.33)
Tacna	17	6.08 (3.54-9.73)	0.19 (0.12 - 0.57)	0.20 (0.06 - 0.62)	2.66 (1.53 - 4.61)	52	15.88 (11.86 - 20.85)	1.70 (1.26 - 2.30	1.18 (0.93 - 1.49)	2.47 (1.84 - 3.31)
Tumbes	73	36.79 (28.88-46.27)	0.13 (0.10 - 0.23)	0.29 (0.19 - 0.45)	1.13 (0.84 - 1.52)	33	16.85 (11.59 - 23.65)	0.13 (0.06 - 0.29	0.14 (0.06 - 0.29)	0.42 (0.03 - 3.64)
Ucayali	231	52.17 (45.7-59.41)	0.24 (0.21 - 0.30)	0.19 (0.15 - 0.24)	0.84 (0.67 - 1.06)	67	16.14 (12.50 - 20.50)	1.17 (0.90 - 1.53)	0.98 (0.78 - 1.24)	1.14 (0.88 - 1.48)

Among adults aged >60 years, hospitalization rates increased in several regions during 2020 and 2021. Notable increases in 2021 were observed in Lambayeque (IRR, 10.02; 95% CI, 7.31–13.74), Piura (IRR, 9.63; 95% CI, 8.61–10.77), Ica (IRR, 6.10; 95% CI, 5.31–7.02), and Huancavelica (IRR, 3.24; 95% CI, 2.79–3.77). In 2022, hospitalization rates remained elevated in San Martín (IRR, 5.19; 95% CI, 4.25–6.33) and Huánuco (IRR, 1.80; 95% CI, 1.50–2.15), whereas decreases were observed in Pasco (IRR, 0.62; 95% CI, 0.44–0.87) and La Libertad (IRR, 0.70; 95% CI, 0.62–0.80) (Table 5).

### 3.7. Mortality rates by region

Pneumonia mortality rates also exhibited significant fluctuations compared to the pre-pandemic period (Fig 4A, 4B). In children <5 years, pre-pandemic mortality was high in Amazonian regions (reaching 3.5 per 100,000 children younger than 5 years), followed by a general decline in 2020 and 2021; however, 2022 showed a severe resurgence in the northern Amazon, with rates again exceeding 3.0–3.5 per 100,000 children younger than 5 years (Fig 4A). In adults aged >60 years, mortality showed substantial regional variation in 2020 but spiked dramatically in 2021, particularly in coastal and central departments, where rates surpassed 70 per 100,000 adults older than 60 years (Fig 4B). By 2022, mortality in the elderly declined to levels closer to the pre-pandemic baseline, although some central regions remained slightly elevated compared to the initial pandemic year.

**Fig 4:**
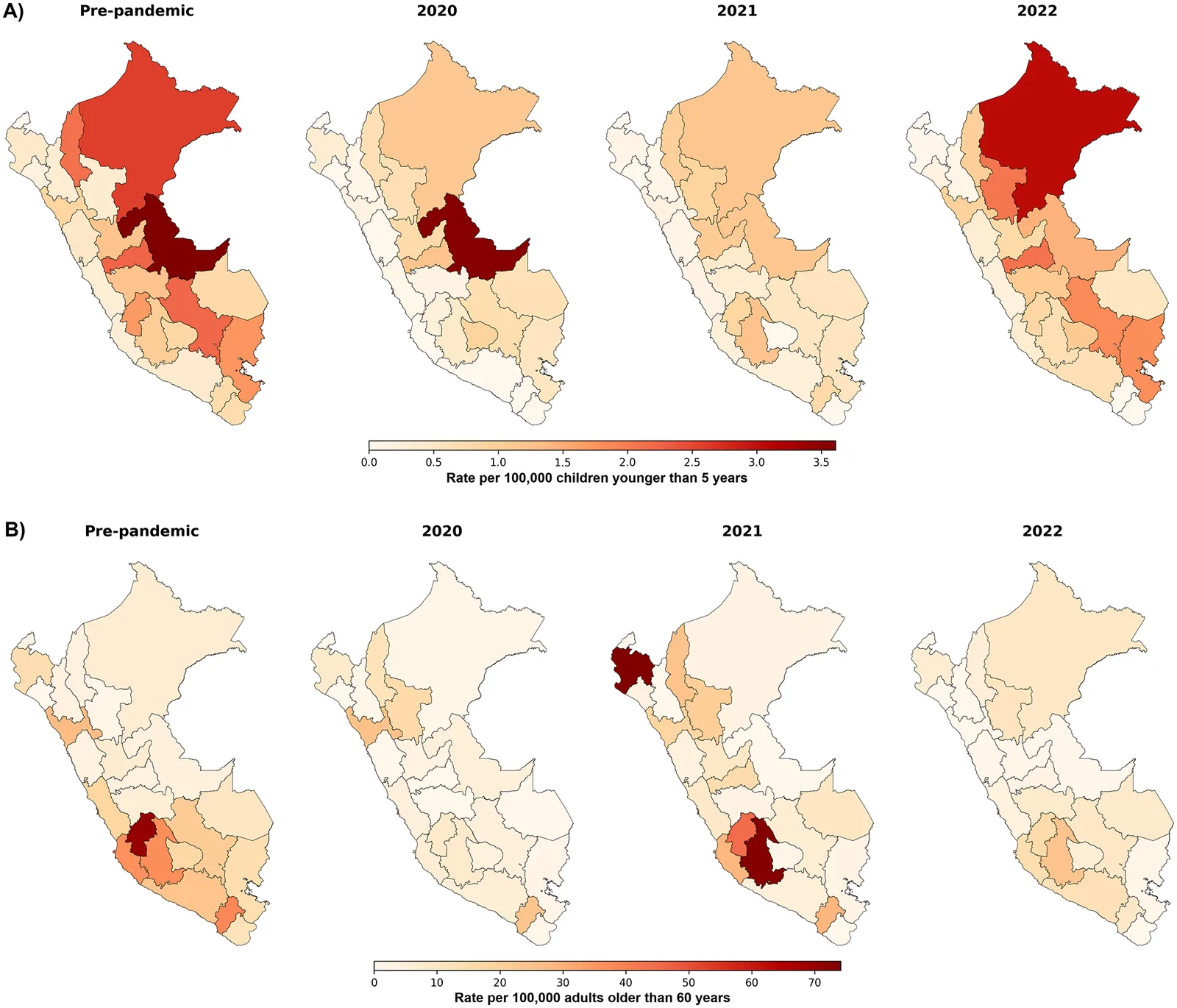
Pneumonia mortality rates in children aged <5 years (A) and adults aged >60 years (B) before and during the COVID-19 pandemic in Peru.

Pneumonia mortality among children younger than 5 years declined in most regions during 2020 and 2021. Marked reductions in 2020 were observed in Junín (IRR, 0.13; 95% CI, 0.02–0.43), La Libertad (IRR, 0.18; 95% CI, 0.04–0.56), and Lima (IRR, 0.23; 95% CI, 0.08–0.54). In 2021, similar reductions were observed in Cusco (IRR, 0.23; 95% CI, 0.10–0.51), Piura (IRR, 0.25; 95% CI, 0.06–0.69), and Junín (IRR, 0.28; 95% CI, 0.09–0.74). In 2022, mortality remained below pre-pandemic levels in several regions, including Piura (IRR, 0.12; 95% CI, 0.01–0.60), although increases were observed in San Martín (IRR, 5.49; 95% CI, 1.78–13.96) (Table 6).

**Table 6:** Pneumonia mortality rates among children aged <5 years and adults aged >60 years, by region (IR, incidence rate per 10,000 children or adults; IRR, incidence rate ratio; CI, confidence interval).

	Mortality rate in children <5 years					Mortality rate in adults >60 years				
	Pre-pandemic (2015–19)		2020	2021	2022	Pre-pandemic (2015–19)		2020	2021	2022
	n (mean)	IR (95% CI)	IRR (95% CI)	IRR (95% CI)	IRR (95% CI)	n (mean)	IR (95% CI)	IRR (95% CI)	IRR (95% CI)	IRR (95% CI)
Amazonas	9	2.12 (0.97 - 4.02)	0.31 (0.06-1.01)	0.47 (0.15-1.26)	0.47 (0.15-1.27)	13	3.69 (1.96-6.31)	3.32 (2.22-4.98)	6.81 (5.29-8.77)	1.54 (0.96-2.45)
Ancash	5	0.47 (0.15 −1.10)	0 (0.0-0.74)	0.23 (0.01-1.29)	0.67 (0.14-2.03)	40	3.25 (2.32-4.43)	0.27 (0.14-0.49)	1.18 (0.85-1.64)	0.29 (0.16-0.54)
Apurimac	4	0.82 (0.22 - 2.10)	1.13 (0.28-4.21)	0 (0.0-0.94)	1.33 (0.33-4.94)	71	16.92 (13.24-21.3)	0.23 (0.13-0.42)	0.1 (0.04-0.23)	0.36 (0.22-0.60)
Arequipa	4	0.39 (0.11 - 0.99)	0 (0.0-0.75)	0.68 (0.14-2.06)	1.69 (0.52-4.36)	378	24.48 (22.07-27.13)	0.24 (0.20-0.29)	0.22 (0.18-0.27)	0.52 (0.46-0.59)
Ayacucho	8	1.05 (0.45 - 2.07)	0.41 (0.09-1.42)	1.15 (0.44-2.70)	0.65 (0.18-1.76)	209	37.05 (32.25-42.5)	0.22 (0.16-0.30)	18.8 (16.08-21.98)	0.67 (0.56-0.81)
Cajamarca	5	0.34 (0.11 - 0.80)	0.21 (0.01-1.17)	0.23 (0.01-1.31)	0.23 (0.01-1.29)	48	3.58 (2.64-4.75)	0.36 (0.21-0.62)	0.14 (0.06-0.32)	0.1 (0.04-0.25)
Callao	4	0.51 (0.14 - 1.31)	0 (0.0-0.73)	0 (0.0-0.94)	0.50 (0.09-1.63)	405	33.39 (30.2-36.87)	0.24 (0.2-0.28)	0.61 (0.53-0.7)	0.21 (0.18-0.26)
Cusco	27	2.23 (1.47 - 3.24)	0.32 (0.15-0.68)	0.23 (0.1-0.51)	0.83 (0.49-1.38)	295	21.96 (19.54-24.61)	0.22 (0.17-0.28)	0.60 (0.48-0.75)	0.46 (0.38-0.56)
Huancavelica	11	1.69 (0.84 - 3.03)	0.15 (0.01-0.82)	0.51 (0.11-1.54)	0.51 (0.11-1.55)	241	70.2 (61.54-79.71)	0.13 (0.09-0.20)	0.63 (0.51-0.77)	0.22 (0.16-0.31)
Huanuco	11	1.21 (0.60 - 2.16)	0.63 (0.23-1.4)	0.89 (0.37-1.89)	0.66 (0.25-1.47)	58	7.66 (5.83-9.92)	0.45 (0.28-0.73)	1.30 (0.97-1.75)	0.2 (0.1-0.40)
Ica	2	0.30 (0.04 - 1.09)	0 (0-1.63)	0.82 (0.11-4.07)	1.26 (0.25-4.43)	323	36.35 (32.55-40.48)	0.19 (0.15-0.25)	0.99 (0.88-1.11)	0.4 (0.34-0.48)
Junin	17	1.20 (0.70 - 1.93)	0.13 (0.02-0.43)	0.28 (0.09-0.74)	0.91 (0.48-1.61)	76	5.97 (4.7-7.48)	0.07 (0.03-0.15)	0.45 (0.32-0.62)	1.36 (1.11-1.66)
La Libertad	15	0.89 (0.50 - 1.47)	0.18 (0.04-0.56)	0.20 (0.05-0.63)	1.08 (0.56-1.98)	537	27.47 (25.19-29.93)	0.93 (0.84-1.02)	0.67 (0.6-0.75)	0.02 (0.01-0.03)
Lambayeque	2	0.19 (0.02 - 0.68	0 (0.0-1.73)	0 (0.0-1.89)	0.94 (0.13-4.69)	10	0.69 (0.33-1.26)	0.41 (0.15-0.97)	9.32 (6.56-13.25)	0.15 (0.02-1.06)
Lima	24	0.30 (0.19 - 0.45)	0.23 (0.08-0.54)	0.43 (0.21-0.77)	0.68 (0.38-1.15)	2069	17.77 (17.01-18.55)	0.27 (0.25-0.29)	0.54 (0.52-0.57)	0.22 (0.21-0.24)
Loreto	28	2.57 (1.71 - 3.71)	0.44 (0.23-0.81)	0.45 (0.25-0.78)	1.19 (0.77-1.80)	53	6.99 (5.24-9.15)	0.22 (0.13-0.37)	0.41 (0.27-0.61)	2.90 (2.26-3.72)
Madre de Dios	1	0.78 (0.02 - 4.36)	0.82 (0.05-4.05)	0.71 (0.04-3.54)	0.69 (0.04-3.42)	9	10.57 (4.83-20.06)	0.06 (0.01-0.44)	1 (0.45-2.21)	0.82 (0.43-1.57)
Moquegua	1	0.75 (0.02 - 4.19)	0 (0.0-2.83)	1.07 (0.07-5.29)	0 (0.0-2.69)	84	38.49 (30.68-47.68)	0.63 (0.46-0.86)	0.75 (0.57-0.99)	0.25 (0.17-0.38)
Pasco	7	2.26 (0.91-4.66)	0.53 (0.16-1.38)	0.19 (0.01-1.04)	0.92 (0.30-2.41)	8	3.48 (1.5-6.86)	0.11 (0.02-0.77)	4.4 (3.2-6.04)	0.81 (0.34-1.9)
Piura	8	0.44 (0.19-0.87)	0.75 (0.28-1.6)	0.25 (0.06-0.69)	0.12 (0.01-0.6)	252	14.21 (12.5-16.11)	0.57 (0.48-0.68)	5.21 (4.87-5.58)	0.48 (0.41-0.57)
Puno	25	1.73 (1.12-2.56)	0.31 (0.11-0.69)	0.35 (0.14-0.73)	1.04 (0.59-1.78	201	14.68 (12.75-16.81)	0.11 (0.07-0.17)	0.17 (0.12-0.25)	0.11 (0.07-0.17)
San Martin	3	0.37 (0.08 - 1.08)	1.73 (0.56-4.40)	2.48 (0.8-6.32)	5.49 (1.78-13.96)	11	1.67 (0.83-2.99)	9.34 (7.07-12.36)	12.41 (9.7-15.89)	6.6 (4.66-9.35)
Tacna	2	0.72 (0.09 - 2.59)	0 (0.0-1.32)	0 (0.0-1.44)	0 (0.0-1.32)	40	12.21 (8.72-16.62)	0.12 (0.05-0.27)	0.03 (0.01-0.12)	0.05 (0.02-0.17)
Tumbes	0	0 (0.00 - 1.51)	— —-—	— —-—	— —-—	6	3.06 (1.12-6.66)	0 (0.0-0.46)	0 (0.0-0.19)	0 (0.0-0.19)
Ucayali	16	3.61 (2.06 - 5.86)	0.98 (0.55-1.74)	0.32 (0.14-0.69)	0.40 (0.18-0.85)	17	4.1 (2.39-6.57)	1.28 (0.76-2.17)	0.09 (0.02-0.36)	0.25 (0.10-0.63)

Among adults aged >60 years, mortality declined in several regions during 2020, including Junín (IRR, 0.07; 95% CI, 0.03–0.15), Puno (IRR, 0.11; 95% CI, 0.07–0.17), and Huancavelica (IRR, 0.13; 95% CI, 0.09–0.20). However, in 2021 substantial increases were observed in Ayacucho (IRR, 18.8; 95% CI, 16.08–21.98), San Martín (IRR, 12.41; 95% CI, 9.7–15.89), and Lambayeque (IRR, 9.32; 95% CI, 6.56–13.25). In 2022, mortality remained below pre-pandemic levels in several regions, including Callao (IRR, 0.21; 95% CI, 0.18–0.26) and Ancash (IRR, 0.29; 95% CI, 0.16–0.54), although increases persisted in San Martín (IRR, 6.6; 95% CI, 4.66–9.35), Loreto (IRR, 2.90; 95% CI, 2.26–3.72), and Junín (IRR, 1.36; 95% CI, 1.11–1.66) (Table 6).

## 4. Discussion

In this nationwide retrospective study, we observed substantial changes in pneumonia epidemiology in Peru during the COVID-19 pandemic compared with the pre-pandemic period. Pneumonia incidence, hospitalizations, and mortality declined markedly among children younger than 5 years, whereas adults aged >60 years experienced increased hospitalizations and mortality. This pattern reveals a clear age-related divergence in pneumonia outcomes, with sustained declines in incidence rates among young children but a disproportionate increase in hospitalizations and deaths among older adults, particularly in 2021. In addition, substantial regional heterogeneity was observed across the country. Together, these findings highlight the complex interplay between pandemic mitigation measures, healthcare access, and underlying population vulnerability in shaping pneumonia dynamics during this period.

Although the marked reduction in pediatric pneumonia incidence and hospitalization rates in 2020 and 2021 occurred during the implementation of non-pharmaceutical interventions such as school closures, mask use, and social restrictions, our ecological study design limits our ability to establish a causal relationship. Similar declines in pediatric respiratory infections have been reported in other settings, together with marked changes in the circulation of respiratory viruses during the COVID-19 pandemic [7,13,18,19]. In our study, this trend was accompanied by a resurgence in 2022, particularly in some regions where hospitalizations exceeded pre-pandemic levels. This pattern may be consistent with increased population susceptibility following a prolonged period of low exposure to respiratory pathogens.

In the United States and Mexico, surveillance data showed substantially reduced influenza and other respiratory virus activity during 2020–2021 compared with previous seasons [18,19]. Similarly, population-based studies have documented reductions in the incidence of respiratory infections in children during the pandemic period, followed by increases after the relaxation of public health measures [7]. The reduction in pneumonia incidence and hospitalizations in Peru among children may also be related to decreased transmission of common respiratory pathogens during the COVID-19 pandemic. However, in Peru, detailed national surveillance data on respiratory pathogens are currently unavailable to directly confirm changes in pathogen circulation. Only one cohort study, limited to a small population, found that detections of human rhinovirus, endemic coronaviruses, and parainfluenza viruses were significantly lower during the COVID-19 pandemic, compared to those in a similar household cohort followed during the pre-pandemic period [20]. Therefore, these findings should be interpreted with caution, as they may also reflect other factors, including changes in healthcare access, health-seeking behavior, or reporting practices during the pandemic. Importantly, Peru’s 13-valent pneumococcal conjugate vaccine (PCV13) immunization has historically reduced childhood pneumonia cases. However, the COVID-19 pandemic caused a 14-percentage-point decrease in pneumococcal coverage [21]. These decreases must be considered alongside viral circulation changes when interpreting shifting pneumonia trends. In addition, disrupted healthcare access and altered parental behavior may have contributed to lower pediatric notifications during 2020–2021. Lockdowns, clinic closures, and fear of nosocomial SARS-CoV-2 exposure may have reduced healthcare-seeking for mild-to-moderate respiratory infections. Therefore, the observed decline may partly reflect changes in healthcare utilization, surveillance, and reporting rather than a true reduction in disease burden. Furthermore, pneumonia mortality among children younger than 5 years was substantially lower than pre-pandemic levels in 2020 and 2021, but returned to levels comparable to the pre-pandemic period by 2022. Sustained decreases may reflect changes in respiratory pathogen circulation, improved hygiene practices, or altered healthcare-seeking behavior among caregivers.

In contrast, adults aged >60 years experienced increases in pneumonia incidence, hospitalizations, and mortality during the pandemic, particularly in 2021. Similar patterns have been reported in settings such as Canada and Switzerland, where older adults bore a disproportionate burden of severe respiratory disease and excess mortality during the COVID-19 pandemic [8,10]. Although some studies have reported reductions in hospitalizations for non-COVID-19 pneumonia, severity and in-hospital mortality among admitted patients remained largely unchanged, especially in older populations [22,23]. Consistent with these findings, a cohort study of adults aged ≥65 years reported a reduction in hospitalizations for non-COVID-19 pneumonia during the pandemic, accompanied by increases in in-hospital mortality (from 10.1% to 18.1%) and 90-day mortality (from 11.6% to 22.3%), highlighting a potential shift toward greater disease severity among hospitalized patients [24]. Several factors may explain these patterns in Peru. Misclassification between COVID-19 and non-COVID-19 pneumonia may have occurred during the early pandemic, when diagnostic capacity was limited. In addition, disruptions in healthcare services and delays in care-seeking may have resulted in more advanced disease at presentation [25]. The high prevalence of comorbidities and age-related immune decline in older adults may have further increased vulnerability to severe outcomes. In addition, a methodological concern is the potential confounding factor of COVID-19. Because the national surveillance dataset did not allow for a definitive microbiological distinction between COVID-19 and other pneumonias, the sharp increases in hospitalizations and mortality among older adults in 2021 may reflect the burden of the pandemic, shifts in cause-of-death reporting, and extreme pressure on the healthcare system rather than a genuine epidemiological shift. Therefore, these results should be interpreted with caution.

Taken together, the observed regional heterogeneity suggests that the impact of the pandemic on pneumonia was not only temporally dynamic but also geographically uneven. Although most regions experienced marked reductions in pneumonia incidence and hospitalizations among children younger than 5 years during 2020 and 2021, some regions showed increases above pre-pandemic levels by 2022. Similarly, among adults aged >60 years, several regions experienced notable increases in pneumonia incidence, hospitalizations, and mortality, particularly in 2021.

These geographic differences may reflect variations in healthcare access, diagnostic capacity, population density, and socioeconomic conditions across regions. In addition, the timing and intensity of COVID-19 waves varied substantially across the country, which may have influenced healthcare utilization and reporting of respiratory infections. Regions in the Amazon and highland areas, where healthcare resources are more limited, may have been particularly vulnerable to disruptions in healthcare services and delays in diagnosis and treatment during the pandemic period. The challenges to the structure of Peru’s healthcare system, which has historically been fragmented, are reflected in these regional disparities, along with distinct geographic and socioeconomic factors. A possible explanation for the dramatic resurgence of pneumonia cases, hospitalizations, and mortality in Amazonian departments like Loreto, Ucayali, and Madre de Dios in 2022 may have been influenced by differences in healthcare infrastructure, access to services, or vaccination coverage; however, these factors were not directly assessed in our study. In contrast, densely populated coastal areas such as Lima and Callao experienced higher initial rates of SARS-CoV-2 transmission but may have differed in diagnostic capacity and healthcare resources. Overall, these findings underscore the need to strengthen surveillance systems, ensure the continuity of immunization programs, and reduce gaps in access to health services, particularly among vulnerable populations.

### Public health implications

Our findings have important implications for public health policy and respiratory infection control in Peru. First, the marked reduction in the incidence, hospitalizations, and mortality from pneumonia in children under five suggests a potential, indirect association with COVID-19 mitigation measures, such as social distancing and school closures. However, because community adherence to these measures and transmission rates were not measured, these findings must be interpreted with caution. These findings are consistent with reduced overall exposure to respiratory pathogens during periods of pandemic restrictions, rather than demonstrating that any single measure was individually effective in reducing childhood pneumonia incidence, hospitalizations, and mortality. In addition, future public health interventions should include the introduction of next-generation immunizations. MINSA Peru has announced new additions to the national immunization program for children under 5 years. In addition, maintaining high vaccination coverage against vaccine-preventable respiratory infections and ensuring continuity of immunization programs during public health emergencies should remain a public health priority.

In contrast, the substantial increase in hospitalizations and mortality from pneumonia in adults over 60 years of age demonstrates the need to strengthen prevention strategies and timely management of respiratory infections in older adults, prioritizing vaccination, access to primary care and continuity of care in contexts of health crises. In this context, the use and coverage of immunization in the elderly population remain a major deficiency in Peru. While the country is facing a rapidly expanding demographic of individuals aged 60 years and older—now approaching 6 million compared to fewer than 400,000 newborns per year—vaccine deployment is profoundly limited by this scale. In this context, strengthening immunization strategies among older adults remains an important public health priority in Peru. Future prevention strategies could consider newer interventions against respiratory infections, including PCV20 and RSV vaccines for older adults, according to national recommendations and availability. Furthermore, regional heterogeneity indicates that public health interventions must be adapted to the epidemiological and structural characteristics of each region, prioritizing those with sustained increases in incidence, hospitalization, or mortality.

Strengthening national surveillance systems is essential to monitor pneumonia trends and detect changes in respiratory pathogen circulation during and after large public health emergencies. Ensuring timely access to diagnosis and treatment for pneumonia remains critical, particularly for older adults who are at higher risk of severe disease and death. In addition, preventive strategies should be reinforced, including vaccination programs against pneumococcus, influenza, and COVID-19, as well as public health measures aimed at improving early healthcare-seeking and access to care among vulnerable populations.

### Limitations

This study has several limitations. First, surveillance data may have been affected by underreporting or changes in reporting practices during the pandemic, especially during periods of healthcare system overload. The national epidemiological surveillance system relies on consistent notifications from primary and secondary healthcare facilities. During the pandemic peaks, the saturation of public hospitals, the reallocation of clinical staff to COVID-19 triage, and the temporary suspension of routine epidemiological reporting units in multiple regions may have contributed to underreporting. The magnitude of this potential underreporting, and whether it differed by age group or region, could not be quantified using the available data. Second, limited diagnostic capacity during the early phases of the pandemic may have led to misclassification between COVID-19–related pneumonia and other pneumonias. Third, the ecological nature of the analysis and the absence of pathogen-specific or individual-level clinical data limited our ability to identify etiologic agents or patient-level risk factors associated with the observed trends. Fourth, mortality data may have been affected by inaccuracies in attributing the cause of death, particularly during the pandemic, when it was difficult to distinguish between primary and contributing causes. These limitations may have introduced potential bias and could have affected the interpretation of the results. Despite these limitations, this study also has several strengths. It uses nationwide surveillance data spanning multiple years before and during the COVID-19 pandemic, allowing a comprehensive assessment of temporal trends in pneumonia incidence, hospitalizations, and mortality across age groups and regions.

## 5. Conclusion

Our findings show that, compared with the pre-pandemic period, the COVID-19 pandemic was associated with a marked reduction in pneumonia incidence, hospitalizations, and mortality among children younger than 5 years in Peru, particularly during 2020 and 2021. In contrast, adults aged >60 years experienced increased hospitalization and mortality rates, with the largest increases observed in 2021. Although pneumonia incidence and hospitalization rates partially returned toward pre-pandemic levels in 2022, substantial geographical heterogeneity persisted across regions.

These findings highlight a differential, age-specific impact of the pandemic on pneumonia burden and suggest that both pandemic-related mitigation measures and disruptions in healthcare access may have contributed to the observed patterns. They also underscore the importance of maintaining robust surveillance systems and implementing targeted prevention and care strategies to protect vulnerable populations, particularly older adults, during future public health emergencies.

## Supporting information

S1 Table STROBE Statement—Checklist. (DOCX)
