# Supplementary material for: Pneumonia incidence, hospitalizations and mortality before and during the COVID-19 pandemic in Peru: An observational study
Source: PLoS One. 2026 Oct 6;21(10):e0351171. doi: 10.1371/journal.pone.0351171 (PMC13641369; doi:10.1371/journal.pone.0351171)
Supplement: Supplementary file 2 [file pone.0351171.s003.docx]

**Manuscript: PONE-D-26-22096 “Incidence, hospitalization, and mortality from pneumonia among children younger than 5 years and adults older than 60 years during the COVID-19 pandemic (2020–22) compared with the pre-pandemic period (2015–19) in Peru: an observational study”**

Please review the following to confirm compliance with the PLOS policy:

The authors have verified the submission content: all submission content generated using AI tools is accurate and valid, none of the content plagiarises other sources, all relevant sources are cited, and all cited sources are relevant. All statements in the article reporting hypotheses, interpretations, results, conclusions, limitations, and implications of the study represent the authors’ own work.

Response: The authors have verified the submission content: all submission content generated using AI tools is accurate and valid, none of the content plagiarises other sources, all relevant sources are cited, and all cited sources are relevant. All statements in the article reporting hypotheses, interpretations, results, conclusions, limitations, and implications of the study represent the authors’ own work.  No generative artificial intelligence (AI) tools were used in the preparation or writing of this manuscript. All statistical analyses were performed using Stata/SE version 17.0.

1. Please note that funding information should not appear in any section or other areas of your manuscript. We will only publish funding information present in the Funding Statement section of the online submission form. Please remove any funding-related text from the manuscript.

Response: This section was removed from the first page.

2. We note that the grant information you provided in the ‘Funding Information’ and ‘Financial Disclosure’ sections do not match.

When you resubmit, please ensure that you provide the correct grant numbers for the awards you received for your study in the ‘Funding Information’ section.

Response: We included “This study was supported by the Universidad Tecnologica del Peru (Grant reference No. P-2025-LIM-26; grant recipient: Dr. Max Carlos Ramírez-Soto) and the PRONII PROCIENCIA - CONACYT – FEEI, Paraguay (Grant recipient: Dr. Christian E. Schaerer and Dr. Diego H. Stalder)”.

3. Thank you for stating the following financial disclosure: “This study was supported by the Universidad Tecnologica del Peru (Grant reference No. P-2025-LIM-26; grant recipient: Dr. Max Carlos Ramírez-Soto) and the PRONII - PROCIENCIA - CONACYT – FEEI, Paraguay (Grant recipient: Dr. Christian E. Schaerer and Dr. Diego H. Stalder).”

Please state what role the funders took in the study. If the funders had no role, please state: "The funders had no role in study design, data collection and analysis, decision to publish, or preparation of the manuscript."

Response: The funders had no role in study design, data collection and analysis, decision to publish, or preparation of the manuscript.

If this statement is not correct you must amend it as needed.

Please include this amended Role of Funder statement in your cover letter; we will change the online submission form on your behalf.

Response: “The funders had no role in study design, data collection and analysis, decision to publish, or preparation of the manuscript” was included in cover letter.

4. Thank you for stating the following financial disclosure:

“This study was supported by the Universidad Tecnologica del Peru (Grant reference No. P-2025-LIM-26; grant recipient: Dr. Max Carlos Ramírez-Soto) and the PRONII - PROCIENCIA - CONACYT – FEEI, Paraguay (Grant recipient: Dr. Christian E. Schaerer and Dr. Diego H. Stalder).”

**Comment:**

We note that one or more of the authors is affiliated with the funding organization, indicating the funder may have had some role in the design, data collection, analysis or preparation of your manuscript for publication; in other words, the funder played an indirect role through the participation of the co-authors. If the funding organization did not play a role in the study design, data collection and analysis, decision to publish, or preparation of the manuscript and only provided financial support in the form of authors' salaries and/or research materials, please do the following:

1. Review your statements relating to the author contributions, and ensure you have specifically and accurately indicated the role(s) that these authors had in your study. These amendments should be made in the online form.

2. Confirm in your cover letter that you agree with the following statement, and we will change the online submission form on your behalf:

Response: The manuscript was funded by our institutions. The funders had no role in study design, data collection and analysis, decision to publish, or preparation of the manuscript. The specific roles of these authors are articulated in the ‘author contributions’ section.

**Comment.** We note that Figures 2-4 in your submission contain map images which may be copyrighted. All PLOS content is published under the Creative Commons Attribution License (CC BY 4.0), which means that the manuscript, images, and Supporting Information files will be freely available online, and any third party is permitted to access, download, copy, distribute, and use these materials in any way, even commercially, with proper attribution. For these reasons, we cannot publish previously copyrighted maps or satellite images created using proprietary data, such as Google software (Google Maps, Street View, and Earth). For more information, see our copyright guidelines: <http://journals.plos.org/plosone/s/licenses-and-copyright>.

Response: Thank you for your comment. We have carefully investigated the provenance and copyright status of the shapefile used to generate the map figures in our manuscript, in accordance with the reviewer's request.

a) Source of the maps and shapefiles:

The departmental boundaries shapefile ("DEPARTAMENTOS.shp") used in our analysis and figures was obtained from the official sources of the Peruvian government. We provide the following evidence to clarify the source:

· The metadata embedded within the shapefile (DEPARTAMENTOS.shp.xml) contains the field <Fuente> with the value "INEI", explicitly identifying the source as the Instituto Nacional de Estadística e Informática (INEI), the national statistical institute of Peru.

· The shapefile was processed using ArcGIS (as noted in the metadata lineage), but the core cartographic data originates from INEI's official geographic databases.

b) Copyright status:

To the best of our knowledge, these maps and shapefiles have not been previously copyrighted in a manner that would restrict their reuse for academic publication. The INEI is a public institution whose mandate includes the open dissemination of statistical and geographic data to support research, public policy, and informed decision-making. The data are made available under a policy of free access and public use, consistent with Peru's transparency and open data frameworks.

c) Permission for CC BY 4.0 license:

Based on the above, we confirm that the shapefile data are not subject to restrictive copyright. The INEI's policy of open access and free reuse permits the publication of maps derived from these data under the Creative Commons Attribution License (CC BY 4.0). We have therefore not sought specific permission, as the source data are publicly and freely available for this purpose. However, to ensure full transparency, we have updated the figure captions in the revised manuscript to include complete source information, as follows:

"The departmental boundary shapefile was sourced from the Instituto Nacional de Estadística e Informática (INEI), Peru. The base map was generated using Python's geopandas and matplotlib."

We trust that this explanation and the updated figure captions adequately address the reviewer's concerns regarding copyright.

**Additional Editor Comments:**

We have identified several major issues that must be addressed before the manuscript can be considered for publication. These relate mainly to:

**Comment 1.** Case definition and classification of pneumonia, including clearer description of notification criteria, possible changes in surveillance and coding over time, and distinction between pneumonia and COVID-19-related events.

Response 1. Thank you for your comment. We included a definition of pneumonia in Methods section “A case of pneumonia was defined based on the clinical and syndromic records compiling information on the notification of acute respiratory infections. Due to the nature of these national registries, specific ICD-10 codes differentiating bacterial, viral (including SARS-CoV-2 infection), or unspecified pneumonia were not available, preventing the explicit exclusion of confirmed cases of COVD-19 from the aggregate counts. Consequently, the national system registers or records outcomes as events. Clinical pneumonia cases secondary to SARS-CoV-2 during the pandemic period (2020–2022) were indistinguishable from non-COVID-19 pneumonia within the national public health dataset”.

**Comment 2.** Potential confounding and misclassification due to COVID-19, which currently is treated as a secondary limitation but has central implications for the interpretation of hospitalization and mortality trends among older adults. Where specific exclusion or identification of COVID-19 cases is not possible, the causal language should be moderated and restricted to changes in events recorded as pneumonia.

Response 2. Thank you for your comment. We have corrected Discussion section.

- “…..Although the marked reduction in pediatric pneumonia incidence and hospitalization rates in 2020 and 2021 occurred during the implementation of non-pharmaceutical interventions such as school closures, mask use, and social restrictions, our ecological study design limits our ability to establish a causal relationship….”
- “…….First, the marked reduction in the incidence, hospitalizations, and mortality from pneumonia in children under five suggests a potential, indirect association with COVID-19 mitigation measures, such as social distancing and school closuresan indirect effect of the non-pharmaceutical interventions implemented in response to COVID-19, such as social distancing, school closures, and mask use. However, because community adherence to these measures and transmission rates were not measured, these findings must be interpreted with caution. They simply suggest that general pandemic restrictions reduced overall exposure, rather than proving that any single measure was individually effective against childhood pneumonia incidence, hospitalizations, and mortality….”.

**Comment 3.** Statistical strategy and time-series analysis, where the current comparison between aggregated pre-pandemic rates and pandemic periods does not account for pre-existing trends, seasonality, autocorrelation, or structural changes in surveillance and healthcare access. We strongly encourage you to consider complementary regression-based analyses (e.g., Poisson or negative binomial models with population offsets, seasonal terms, and regional effects), as well as sensitivity analyses distinguishing 2020 and 2021.

Response 3: Thank you for your comment. Although weekly data were available, our objective was not to develop a predictive or explanatory time-series model, but rather to compare the magnitude of epidemiological changes across predefined pandemic periods. Weekly plots were included for descriptive purposes to illustrate temporal trends and seasonal patterns, whereas the primary estimates were based on comparisons between periods using incidence rate ratios. We acknowledge that regression models incorporating seasonal terms or interrupted time-series analyses could provide additional insights. However, these approaches address a different analytical objective and would require explicit modeling of temporal dependence, which was beyond the scope of the present study. We have acknowledged this consideration in the Limitations section.

**Comment 4.** Numerical and formatting inconsistencies in Tables 1 and 2, which need thorough verification against the analytical dataset and correction to ensure that all values are aligned with the appropriate age group, year, outcome, and region.

Response 4. Thank you for your comment. We have corrected Results section and Table 1. We have made sure to review the text of the results. Furthermore, for better understanding by the readers, we have divided Table 2 into three tables: Table 2. Incidence; Table 3. Hospitalization; and Table 4. Mortality.

**Comment 5.** Clarification of mortality outcomes, denominators, and age-group definitions, including how deaths are classified in SINADEF, whether COVID-19-related pneumonia deaths are included or indistinguishable, and the exact age ranges used for children and older adults.

**Response 5.** Thank you for your comment. We thank the reviewer for this valuable comment. First, we have clarified that the incidence, hospitalization, and mortality rates were calculated using age-specific population projections from the National Institute of Statistics and Informatics of Peru (Instituto Nacional de Estadística e Informática [INEI]) as the denominator and were expressed per 100,000 population. The age groups corresponded to children aged <5 years (0–4 years) and older adults aged ≥60 years, in accordance with the classification used in the official databases of the Peruvian Ministry of Health. Mortality data were obtained directly from the aggregated database published by the National Center for Epidemiology, Prevention and Disease Control of Peru (CDC Peru), which compiles the official mortality information reported through the national Acute Respiratory Infections (ARI) surveillance system, including deaths associated with both pneumonia and COVID-19. This clarification has been incorporated into the Methods section.

**Reviewers' comments:**

**Reviewer #1:**

The study is timely, relevant, and generally well structured. One of the major strengths of the study is the inclusion of national-level longitudinal data from 2015 to 2022, allowing comparison between pre-pandemic, pandemic, and post-pandemic periods. The age-stratified and region-specific analyses substantially improve the epidemiological interpretation of the findings. The results clearly demonstrate contrasting patterns between pediatric and older adult populations, with marked declines in pneumonia incidence and hospitalization among children and increased hospitalization and mortality among older adults during the pandemic period.

The statistical approach based on incidence rates and incidence rate ratios using Poisson assumptions is generally appropriate for this type of surveillance analysis. However, several methodological aspects should be clarified or strengthened:

**Comment 1.** The manuscript would benefit from a more detailed explanation regarding the potential impact of underreporting and healthcare access disruptions during the COVID-19 pandemic. Reductions in healthcare utilization may have substantially influenced notification rates, particularly among children.

Response 1. Thank you for your comment. We have improved the Discussion section with your feedback.

- ….In addition, disrupted healthcare access and altered parental behavior drove down pediatric notifications during 2020–2021. Lockdowns, clinic closures, and fear of nosocomial SARS-CoV-2 exposure prevented families from seeking care for mild-to-moderate infections. These unrecorded cases show that the reported decline may reflect a breakdown in surveillance and access, rather than a true drop in disease burden.
- Limitation. The national epidemiological surveillance system relies on consistent notifications from primary and secondary healthcare facilities. During the pandemic peaks, the saturation of public hospitals, the reallocation of clinical staff to COVID-19 triage, and the temporary suspension of routine epidemiological reporting units in multiple regions inevitably led to substantial underreporting. This disruption particularly affected the pediatric population because healthcare resources were aggressively prioritized toward adult emergency departments.

**Comment 2.** Although the authors discuss regional heterogeneity, additional analyses exploring potential determinants of these regional differences (e.g., healthcare infrastructure, COVID-19 burden, vaccination coverage, altitude, or socioeconomic conditions) would strengthen the epidemiological interpretation.

Response 2. Thank you for your comment. We have improved the Discussion section with your feedback “The challenges to the structure of Peru's healthcare system, which has historically been fragmented, are reflected in these regional disparities, along with distinct geographic and socioeconomic factors. A possible explanation for the dramatic resurgence of pneumonia cases, hospitalizations, and mortality in Amazonian departments like Loreto, Ucayali, and Madre de Dios in 2022 likely stems from severe infrastructure deficits or lower vaccination coverage. In contrast, densely populated coastal areas such as Lima and Callao experienced higher initial rates of SARS-CoV-2 transmission but had greater diagnostic capacity and hospital flexibility to absorb the healthcare strain”.

**Comment 3.** Some inconsistencies appear in the interpretation of incidence rate ratios among older adults. For example, Table 1 reports lower IRRs despite crude annual rates appearing numerically similar or higher in some years. The authors should clarify the exact calculation strategy for pooled pre-pandemic denominators and IRRs to improve transparency.

Response 3. Thank you for your comment. We have corrected Results section and Table 1. We have made sure to review the text of the results. Furthermore, for better understanding by the readers, we have divided Table 2 into three tables: Table 4. Incidence; Table 5. Hospitalization; and Table 6. Mortality. We have provided additional details regarding the statistical analyses in the Methods section.

**Reviewer #2:**

**Major comments**

**Comment 1.** Case definition and classification of pneumonia. The manuscript should clarify more precisely how pneumonia was defined in the surveillance system used. It is not sufficiently clear whether the cases represent clinical pneumonia, radiological pneumonia, hospital pneumonia, community acquired pneumonia, severe pneumonia, acute respiratory syndrome, or administrative notification categories. This definition is central to the interpretation of the results, particularly during the pandemic period, when COVID 19 may have been recorded as pneumonia or as an associated cause. I suggest that the authors include a detailed description of the notification criteria used by CDC Peru, distinguishing cases, hospitalizations, and deaths. It is also important to explain whether there were any changes in case definition, notification flow, diagnostic coding, or the surveillance system between 2015 and 2022.

Response 1. Thank you for your comment. We included a definition of pneumonia in Methods section “A case of pneumonia was defined based on the clinical and syndromic records compiling information on the notification of acute respiratory infections. Due to the nature of these national registries, specific ICD-10 codes differentiating bacterial, viral (including SARS-CoV-2 infection), or unspecified pneumonia were not available, preventing the explicit exclusion of confirmed cases of COVD-19 from the aggregate counts. Consequently, the national system registers or records outcomes as events. Clinical pneumonia cases secondary to SARS-CoV-2 during the pandemic period (2020–2022) were indistinguishable from non-COVID-19 pneumonia within the national public health dataset”.

**Comment 2.** Potential confounding by COVID 19. The main interpretative limitation of the study is the distinction between COVID 19 associated pneumonia and pneumonia from other causes. The authors acknowledge the possibility of misclassification, but this issue should be treated as a central methodological concern rather than as a secondary limitation. The large increase in hospitalization and mortality among adults older than 60 years in 2021 may partly reflect the burden of COVID 19, changes in cause of death reporting, or healthcare system pressure, rather than an actual increase in non COVID 19 pneumonia.

The authors should clarify whether the database allows confirmed COVID 19 cases to be excluded, whether specific ICD codes can be identified, or whether viral, bacterial, and unspecified pneumonia can be distinguished. If this distinction is not possible, the causal language should be moderated, and statements suggesting a true change in pneumonia epidemiology should be replaced by more cautious wording, such as changes in notifications, hospitalizations, and deaths recorded as pneumonia.

**Response 2.** Thank for you for your comment. We have corrected Method section “Due to the nature of these national registries, specific ICD-10 codes differentiating bacterial, viral (including SARS-CoV-2 infection), or unspecified pneumonia were not available, preventing the explicit exclusion of confirmed cases of COVD-19 from the aggregate counts. Consequently, the national system registers or records outcomes as events”. We also have corrected Discussion section. “In addition, a methodological concern is the potential confounding factor of COVD-19. Because the national surveillance dataset did not allow for a definitive microbiological distinction between COVD-19 and other pneumonias, the sharp increases in hospitalizations and mortality among older adults in 2021 may reflect the burden of the pandemic, shifts in cause-of-death reporting, and extreme pressure on the healthcare system rather than a genuine epidemiological shift. Therefore, these results should be interpreted with caution”.

**Comment 3.** Statistical strategy. The comparison of the pandemic period with an average or aggregated rate from 2015 to 2019 is simple and understandable, but limited for a national time series. The analysis does not control for previous trends, seasonality, autocorrelation, structural regional differences, demographic changes, or abrupt disruptions in the surveillance system. For a study focused on the impact of the pandemic, an interrupted time series approach, Poisson or negative binomial regression with a population offset, seasonal terms, and regional effects would substantially strengthen the inferences.

I suggest that the authors consider at least a complementary analysis using regression models including year, epidemiological week, pandemic period, region, and age group. A sensitivity analysis excluding 2020 or treating 2020 and 2021 separately would also be useful, since these two years had different pandemic and healthcare patterns.

Response 3. Thank you for your comment. We thank the reviewer for this valuable comment. In response, we performed additional analyses using generalized linear models (GLMs) with a Poisson distribution and log link, incorporating the logarithm of the population as an offset, adjusting for epidemiological week to account for seasonality and for geographic region to account for spatial heterogeneity, and using robust standard errors. The results were consistent with the incidence rate ratios estimated in the primary analysis, supporting the robustness of our findings. We acknowledge, however, that this approach does not explicitly model temporal autocorrelation or pre-existing trends, as would be achieved with an interrupted time-series analysis. Our objective was to compare the observed rates during the pandemic period with those of the pre-pandemic period using a population-based epidemiological approach, rather than to estimate causal effects through specialized time-series models. This limitation has been acknowledged in the Limitations section of the manuscript.

**Comment 4.** Excessive causal interpretation. In several sections, the manuscript attributes the reduction in pediatric pneumonia to non pharmaceutical interventions, such as school closures, mask use, and physical distancing. This hypothesis is plausible, but the ecological design does not allow causality to be established. In addition, the decline in notifications may reflect reduced healthcare seeking, limited access to care, underreporting, or diversion of surveillance efforts toward COVID 19.

I recommend that the discussion clearly distinguish plausible explanations from conclusions demonstrated by the data. The sentence on the usefulness of non pharmaceutical interventions to control pediatric respiratory infections should be softened, as the study did not measure adherence to these measures or viral.

Response 4. Thank you for your comment. We have corrected Discussion section.

- “…..Although the marked reduction in pediatric pneumonia incidence and hospitalization rates in 2020 and 2021 occurred during the implementation of non-pharmaceutical interventions such as school closures, mask use, and social restrictions, our ecological study design limits our ability to establish a causal relationship….”
- “…….First, the marked reduction in the incidence, hospitalizations, and mortality from pneumonia in children under five suggests a potential, indirect association with COVID-19 mitigation measures, such as social distancing and school closuresan indirect effect of the non-pharmaceutical interventions implemented in response to COVID-19, such as social distancing, school closures, and mask use. However, because community adherence to these measures and transmission rates were not measured, these findings must be interpreted with caution. They simply suggest that general pandemic restrictions reduced overall exposure, rather than proving that any single measure was individually effective against childhood pneumonia incidence, hospitalizations, and mortality….”.

**Comment 5. Inconsistencies and problems in Table 1.** Table 1 appears to have column misalignment, especially in the rows on hospitalizations and deaths. Some values seem to be displaced between children and adults, years, and IRRs. This compromises the reliability of the interpretation of the results. For example, some IRR and rate values appear to be in positions that are incompatible with the text. The authors should carefully revise the formatting of Table 1 and ensure that each value corresponds to the correct age group, year, and outcome.

I recommend rebuilding the table with simpler columns: age group, outcome, pre pandemic period, 2020, 2021, 2022, IR, IRR, and 95% CI. A supplementary version with absolute numbers would also be helpful.

Response 5. Thank you for your comment. We have corrected Results section and Table 1 “Among adults aged >60 years, incidence increased by 3% in 2020 (IRR, 1.03; 95% CI, 1.01–1.05) and 2022 (IRR, 1.03; 95% CI, 1.01–1.05), and showed a marked increase of 51% in 2021 (IRR, 1.51; 95% CI, 1.48–1.54) (Table 1).”

**Comment 6. Inconsistencies and possible errors in Table 2.** Table 2 is extensive and contains multiple apparent typographical, formatting, and possibly calculation errors. There is a title error, “Incidence rate in children in adults >60 years”, as well as “Hopitalization”. Some cells appear to have missing parentheses, incomplete intervals, or biologically and statistically unexpected values. There are also discrepancies between the text and the table, such as the mention of Puno with an IRR of 3.60 for incidence among adults, while the table appears to show another value for Puno and a high value for San Martín.

This table should be reviewed in full. I recommend that the authors verify all regional estimates directly from the analytical dataset and preferably provide a complete supplementary table. In the main text, it would be better to highlight only the main regional findings, supported by more legible maps or figures.

Response 5. Thank you for your comment. We have corrected Results section and Table 2. We have made sure to review the text of the results. Furthermore, for better understanding by the readers, we have divided Table 2 into three tables: Table 4. Incidence; Table 5. Hospitalization; and Table 6. Mortality.

**Comment 7.** Mortality outcomes. Pneumonia mortality during the pandemic is particularly susceptible to attribution bias. The manuscript should explain whether deaths were classified by underlying cause, direct cause, or associated cause in SINADEF. It should also state whether deaths from COVID 19 with pneumonia were included, excluded, or indistinguishable.

Without this information, the interpretation of increased mortality among older adults should be very cautious. The statement that pneumonia mortality increased may be acceptable as a description of recorded deaths, but not as evidence of an increase in non COVID 19 pneumonia.

Response 7: Thank you for your comment. We have corrected Methods section “Pneumonia-related mortality data extracted from SINADEF are classified based on the underlying cause of death. No additional information was available on the website of the CDC Peru. Due to the nature of these national registries, specific ICD-10 codes differentiating bacterial, viral (including SARS-CoV-2 infection), or unspecified pneumonia were not available, preventing the explicit exclusion of confirmed cases of COVD-19 from the aggregate counts. Consequently, the national system registers or records outcomes as events”.

**Comment 8.** Population denominator and age groups. The manuscript uses “children under five” and “adults older than 60 years”, but in some sections it alternates between “aged >60 years” and “adults older than 60 years”. It should be clear whether the adult group includes individuals aged 60 years or only those older than 60 years, that is, 61 years and older. This distinction may affect the population denominators. Similarly, it should be clarified whether children younger than 5 years include children aged 0 to 4 completed years.

Comment 8. Thank you for your comment. We have corrected Method section “We also included pneumonia-related hospitalizations in children under 5 years (include children aged 0 to 4 completed years) and adults older than 60 years (include adults aged 61 years and older)…”.

**Comment 9.** The term “post pandemic”. The manuscript refers to 2022 as the “post pandemic” period. This terminology is debatable, since SARS-CoV-2 circulation, healthcare impact, and epidemic waves were still present in many countries in 2022. I suggest replacing this term with “period of relaxation of pandemic measures”, “transition year”, or simply “2022”, avoiding the implication that the pandemic had ended.

Comment 9. Thank you for your comment. Figure 1 has been corrected.

**Comment 10. Data availability and reproducibility.** The data availability statement provides only the link to the public database. It would be desirable for the authors to make available the final analytical dataset, variable dictionary, statistical code used in Stata, and data cleaning scripts. This would increase transparency and allow verification of the IR and IRR calculations, especially given the inconsistencies observed in the tables.

Comment 10. Thank you for your comment. The information will be available at the following link: <https://data.mendeley.com/preview/mgfsrxpvsd?a=ece66b5e-736b-494e-8b73-bd5ce0fb5498>

**Comment 11. Ethics.** The ethics justification is appropriate in principle, as the study uses aggregated, public, and anonymized data. However, the authors should confirm whether Peruvian local regulations waive formal ethics review for analyses of aggregated public data. If an exemption letter or institutional consultation exists, this should be reported.

Comment 11. Thank you for your comment. We have included a sentence in 2.4. Ethic section “Under local regulations, formal ethics review is waived for analyses of such public, non-identifiable data”.

**Comment 12. Discussion and public health policy.** The public health implications section is relevant, but it could be more specific. The manuscript recommends surveillance, vaccination, and timely access to care, but it does not discuss which vaccines would be priorities in the Peruvian context, nor does it differentiate strategies for children and older adults. The mention of RSV vaccination in children and adults should be contextualized cautiously, since the analyzed data do not include specific viral etiology and do not measure RSV.

Response 12. Thank you for your comment. We have included two sentences in Discussion and public health policy section.

1. In addition, future public health interventions should include the introduction of next-generation immunizations. MINSA Peru has announced new additions to the national immunization program for children under 5 years. The new additions include the PCV20 and meningococcal vaccines, as well as the use of monoclonal antibodies for RSV in newborns.
2. In this context, the use and coverage of immunization in the elderly population remain a major deficiency in Peru. While the country is facing a rapidly expanding demographic of individuals aged 60 years and older—now approaching 6 million compared to fewer than 400,000 newborns per year—vaccine deployment is profoundly limited by this scale. The annual influenza vaccine coverage remains at a very low level in older Peruvians, and the introduction of modern essential interventions, such as the adult RSV vaccine or a PCV20 booster dose, has not yet occurred in this vulnerable population.

**Minor comments**

**Comment 1.** The title is long and repetitive. I suggest shortening it to improve clarity. For example: “Pneumonia incidence, hospitalizations and mortality before and during the COVID 19 pandemic in Peru: a nationwide observational study”.

Response 1. Thank you for your comment. The title has been corrected “Pneumonia incidence, hospitalizations and mortality before and during the COVID 19 pandemic in Peru: a nationwide observational study”.

**Comment 2.** The abstract should use “death rates” or “mortality rates”, but not “deaths rates”.
The study design is described as a “cross sectional retrospective observational study” in the abstract, but as a “retrospective observational study” in the methods. This should be standardized. For aggregated time series data, “retrospective population based time series study” may be more appropriate.

Response 2. Thank you for your comment. The Design has been corrected “retrospective population-based time series study”.

**Comment 3.** There are recurrent grammar issues in English, such as “habitants” instead of “inhabitants” or “population”, “deaths rates”, and inconsistencies in singular and plural usage.
The term “incidence risk ratios” should be avoided if the authors are calculating “incidence rate ratios”. Risk and rate are different concepts.

Response 3. Thank you for your comment. We have improved the manuscript with your feedback, including the legends of Figures.

**Comment 4.** The authors should clearly report whether the confidence intervals for the IRRs were calculated using an exact method, normal approximation, Poisson regression, or another approach.

Response 3. Thank you for your comment. We have clarified the method used to estimate incidence rate ratios (IRRs) and their corresponding confidence intervals in the Statistical Analysis section. In the descriptive analysis, IRRs were calculated by comparing the observed rates during the pandemic period with those of the pre-pandemic period, and their 95% confidence intervals were estimated using the exact method for Poisson rates. As a complementary sensitivity analysis, we fitted generalized linear models (GLMs) with a Poisson distribution and log link, incorporating the logarithm of the population as an offset, adjusting for epidemiological week and geographic region, and using robust standard errors. In these models, the 95% confidence intervals were derived from the Poisson regression model. Both approaches produced consistent estimates in terms of the direction and magnitude of the observed effects, supporting the robustness of our findings.

**Comment 5.** I recommend including a case fatality analysis, such as deaths per reported cases or deaths per hospitalizations, with all necessary caveats. This would help interpret whether the increase among older adults reflects greater severity among notified cases or an absolute increase in events.

Response 5. Thank you for your comment. To assess whether the observed increase in mortality, particularly among older adults, reflected greater severity among reported pneumonia cases in addition to changes in the absolute number of events, we performed an additional case-fatality analysis using generalized linear models (GLMs) with a Poisson distribution and log link. To estimate mortality among reported pneumonia cases, the annual number of pneumonia deaths was used as the dependent variable, and the logarithm of the annual number of reported pneumonia cases was included as an offset. Models were additionally adjusted for epidemiological week and geographic region, and robust standard errors were used (Table 2). Similarly, mortality among hospitalized pneumonia patients was estimated using the logarithm of the annual number of hospitalizations as the offset (Table 3).

**Reviewer #3:**

Here is a list of specific comments. Note: line and page numbering in reviews and comments is based on ruler applied in Editorial Manager-generated PDF.

**Comment 1.** Page 5, line 136: I recommend clarifying in the manuscript whether hospitalizations and deaths are pneumonia-related or all-cause. [Please read Comment #3 below before responding.]

Response 1. Thank you for your comment. We included a definition of pneumonia in Methods section “A case of pneumonia was defined based on the clinical and syndromic records compiling information on the notification of acute respiratory infections. Due to the nature of these national registries, specific ICD-10 codes differentiating bacterial, viral (including SARS-CoV-2 infection), or unspecified pneumonia were not available, preventing the explicit exclusion of confirmed cases of COVD-19 from the aggregate counts. Consequently, the national system registers or records outcomes as events. Clinical pneumonia cases secondary to SARS-CoV-2 during the pandemic period (2020–2022) were indistinguishable from non-COVID-19 pneumonia within the national public health dataset”.

**Comment 2.** Page 5, lines 145–146: I recommend clarifying in the manuscript what non-pneumonia cases refer to. Also, please clarify “cases” in line 151. [Please read Comment #3 below before responding.]

Response 2. Thank you for your comment. 'Non-pneumonia cases' referred to other types of respiratory infections recorded in the National Surveillance System. To avoid confusion, the term 'non-pneumonia' has been removed from the manuscript.”

**Comment 3.** Page 5, lines 152–160: This paragraphs address both Comments 1 and 2 above. I recommend clarifying them at their first appearances.

Response 3. Thank you for your comment. We included a definition of pneumonia in Methods section “A case of pneumonia was defined based on the clinical and syndromic records compiling information on the notification of acute respiratory infections. Due to the nature of these national registries, specific ICD-10 codes differentiating bacterial, viral (including SARS-CoV-2 infection), or unspecified pneumonia were not available, preventing the explicit exclusion of confirmed cases of COVD-19 from the aggregate counts. Consequently, the national system registers or records outcomes as events. Clinical pneumonia cases secondary to SARS-CoV-2 during the pandemic period (2020–2022) were indistinguishable from non-COVID-19 pneumonia within the national public health dataset”.

**Comment 4.** Page 6, lines 162–164: I recommend clarifying the descriptions of the outcomes. Pneumonia incidence, pneumonia-related hospitalization rate, and pneumonia-related mortality rate are the outcomes, whereas the change in these measures between the pre-pandemic and pandemic periods is the parameter of interest.

Response 4. Thank you for your comment. We have improved the 2.2. Outcome section with your feedback. “*The primary outcomes evaluated in this study were age-specific was the change of pneumonia incidence, hospitalization, and mortality rates per 100,000 inhabitants during and after the COVID-19 pandemic (2020–22), compared with the pre-pandemic period (2015–19). The parameters of interest were the relative changes in these measures between the pre-pandemic period (2015–19) and the pandemic/post-pandemic periods (2020–22)*”.

**Comment 5.** Page 6, line 163: I recommend clarifying the inhabitants information comes from (i.e., the denominators of the rates) in Section 2.1.

Response 5. Thank you for your comment. We have improved the Section 2.1 with your feedback “…..*To obtain the denominators for the rate calculations, annual regional population estimates and projections for children younger than 5 years and adults older than 60 years for the 2015–2022 period were obtained from the National Institute of Statistics and Informatics of Peru (INEI) [17]*”.

**Comment 6.** Page 7, line 185: I recommend clarifying the 95% CIs for IRRs are also exact 95% CIs.

Response 6. Thank you for your comment. We have improved the Section 2.3 with your feedback *“…..Exact 95% CIs for the estimated incidence rate ratios (IRRs), defined as incidence risk ratios for pneumonia cases, hospitalizations, and deaths, were calculated…..*”.

**Reviewer #4:**

This is a well written manuscript describing the impact of the early years of the COVID-19 pandemia in Peru on the incidence, hospitalizations and deaths attributable to pneumonia by the Peruvian national health system. This reviewer recommends its publication, after considering the following minor observations:

**Minor Observations:**

**Comment 1.** The introduction seems a bit long. It is up to the editors to consider if it needs or not to be shortened.

Response 1. Thank you for your comment. While the Introduction is somewhat lengthy, it helps to understand the context of this and provides a clear justification. We believe its length should be maintained.

**Comment 2.** Lines 148 and 159 where it says “…of the MINSA”, it should say of MINSA.

Response 2. Thank you for your comment. The error was corrected.

**Comment 3.** The author cites (ref 17) an old publication of the Peruvian National Institute of Statistics and Informatics. Peru has conducted a recent (2025) national census which may provide better denominators for the study analysis as compared with old population projections. This may be important, because the fertility in Peru is dropping rapidly, while the population 60yrs and older is growing rapidly.

Response 3. Thank you for your comment. The updated reference is as follows: National Institute of Statistics and Informatics. 2017 National Censuses: XII Population, VII Housing, and III Indigenous Communities - REDATAM Data Query System [Internet]. Lima: INEI; 2017 [cited 2026 Jun 29]. Available from: <https://censos2017.inei.gob.pe/redatam/>. This reference shows the projections for all study years from 2015 to 2022. The 2025 survey does not include projections for the study.

**Comment 4.** Fig 1 in the submitted manuscript is blurred. It should be published with very clear text.

Response 4. Thank you for your comment. Figure 1A-F was uploaded in high resolution. It can be downloaded in TIFF format.

**Comment 5.** The authors are correct that the study data, given all mentioned potential factors that may affect its precision, should be taken with caution. However, the important changes by surveillance years, and regions, are important observations, documenting the need to address older populations as well as regions with poor access to health care.

Response 5. Thank you for your comment.

**Comment 6.** The authors mentioned that no studies have been conducted in Peru evaluating the impact of the COVID-19 pandemic on child respiratory morbidity. The authors should review this reference as an example. Others may exist as well. Howard LM, Gil AI, Ecker L, Chen H, Chen Q, Cornejo R, Rios S, Ochoa M, Peña B, Flores O, Lanata CF, Grijalva CG. Declines in human rhinovirus, coronavirus, parainfluenza, and adenovirus infections during the COVID-19 pandemic: Evidence from household-based cohort studies in Lima, Peru. Influenza and Other Respiratory Viruses, 2026;20:e70233. doi: 10.1111/irv.70233.

Response 6. Thank you for your comment. We have included the reference doi: 10.1111/irv.70233. However, the study is limited to a small population “…….However, in Peru, detailed national surveillance data on respiratory pathogens are currently unavailable to directly confirm changes in pathogen circulation. Only one cohort study, limited to a small population, found that detections of human rhinovirus, endemic coronaviruses, and parainfluenza viruses were significantly lower during the COVID-19 pandemic, compared to those in a similar household cohort followed during the pre-pandemic period……….”.

**Comment 7.** An important factor not analyzed by the authors is the correlation between ARI/pneumonia rates and vaccines used in Peru in children <5yrs. Particularly, as an example, the change from pneumo-7 to pneumo-13 conjugated vaccines. Vaccine coverage may also be another important factor, as documented in this reference: Silva LAN, Costa FS, Cata-Preta BO, Huicho L, Lanata CF, Araujo MAM, Ochoa TJ, Mengistu T, Hogan D, Barros AJD, Victora CG. The impact COVID-19 pandemic on coverage and inequalities in childhood immunization in Peru. Int J Equity Health 2025 May 15;24(1):138. doi: 10.1186/s12939-025-02505-3. PMID: 40375225.

Response 7. Thank you for your comment. We have included the reference doi: 10.1186/s12939-025-02505-3 in Discussion section “…..Importantly, Peru's 13-valent pneumococcal conjugate vaccine (PCV13) immunization has historically reduced childhood pneumonia cases. However, the COVID-19 pandemic caused a 14-percentage-point decrease in pneumococcal coverage [25]. These decreases must be considered alongside viral circulation changes when interpreting shifting pneumonia trends…..”.

**Comment 8.** When discussing policy implications, in addition to access to care and appropriate clinical management of ALRI cases, the introduction of new vaccines are important interventions. For children <5yrs, Peru has just announced the introduction of pneumo-20 conjugated vaccines, meningococcal vaccines, and the use of monoclonal antibodies in neonates for RSV. However, immunization use and coverage in elderly population is a major deficiency in Peru. For instance, the coverage of annual doses of the influenza vaccine is very low in Peruvians 60yrs and older. The RSV vaccine has not yet been introduced. And the booster dose of a pneumo-20 conjugated vaccine has also not been introduced in this vulnerable population. In part given the number of doses required (Peru is approaching 6 million population size of 60yrs and older, compared to less than 400,000 newborns per year).

Response 8. Thank you for your comment. We included two paragraphs in the Discussion section - Public health implications:

- ……..In addition, future public health interventions should include the introduction of next-generation immunizations. MINSA Peru has announced new additions to the national immunization program for children under 5 years. The new additions include the PCV20 and meningococcal vaccines, as well as the use of monoclonal antibodies for RSV in newborns.
- …….. In this context, the use and coverage of immunization in the elderly population remain a major deficiency in Peru. While the country is facing a rapidly expanding demographic of individuals aged 60 years and older—now approaching 6 million compared to fewer than 400,000 newborns per year—vaccine deployment is profoundly limited by this scale. The annual influenza vaccine coverage remains at a very low level in older Peruvians, and the introduction of modern essential interventions, such as the adult RSV vaccine or a PCV20 booster dose, has not yet occurred in this vulnerable population.
